# Genomic insights into Neolithic founding paternal lineages around the Qinghai-Xizang Plateau using integrated YanHuang resource

**DOI:** 10.1016/j.isci.2024.111456

**Published:** 2024-11-22

**Authors:** Mengge Wang, Yunhui Liu, Lintao Luo, Yuhang Feng, Zhiyong Wang, Ting Yang, Huijun Yuan, Chao Liu, Guanglin He

**Affiliations:** 1 Institute of Rare Diseases, West China Hospital of Sichuan University, Sichuan University, Chengdu 610000, China; 2 Center for Archaeological Science, Sichuan University, Chengdu 610000, China; 3 Department of Forensic Medicine, College of Basic Medicine, Chongqing Medical University, Chongqing 400331, China; 4 Anti-Drug Technology Center of Guangdong Province, Guangzhou 510230, China

**Keywords:** Evolutionary biology, Paleobiology, Anthropology

## Abstract

Indigenous populations of the Qinghai-Xizang Plateau exhibit unique high-altitude adaptations, especially within Tibeto–Burman (TB) groups. However, the paternal genetic heritage of eastern Plateau regions remains less explored. We present one integrative Y chromosome dataset of 9,901 modern and ancient individuals, including whole Y chromosome sequences from 1,297 individuals and extensive Y-SNP/STR genotype data. We reveal the Paleolithic common origin and following divergence of Qinghai-Xizang Plateau ancestors from East Asian lowlands, marked by subsequent isolation and Holocene expansion involving local hunter-gatherers and millet-farming communities. We identified two key TB-related founding lineages, D-Z31591 and O-CTS4658, which underwent significant expansions around 5,000 years ago on the Qinghai-Xizang Plateau and its eastern Tibetan-Yi Corridor. The genetic legacy of these TB lineages highlights crucial migration pathways linking the Plateau and lowland southwestern China. Our findings align paternal genetic structures with East Asian geography and linguistic groups, underscoring the utility of Y chromosome analyses in unraveling complex paternal histories.

## Highlights

• We reported an integrative Yanhuang cohort Y chromosome dataset from 9,901 individuals
• Paleolithic and Holocene events shaped Qinghai-Xizang Plateau paternal lineages
• Two Tibeto-Burman-related founding lineages had star-like expansions ∼5,000 years ago
• We revealed migration corridors and shared paternal origins among Sino-Tibetan-speaking populations

## Graphical abstract

**Figure:**
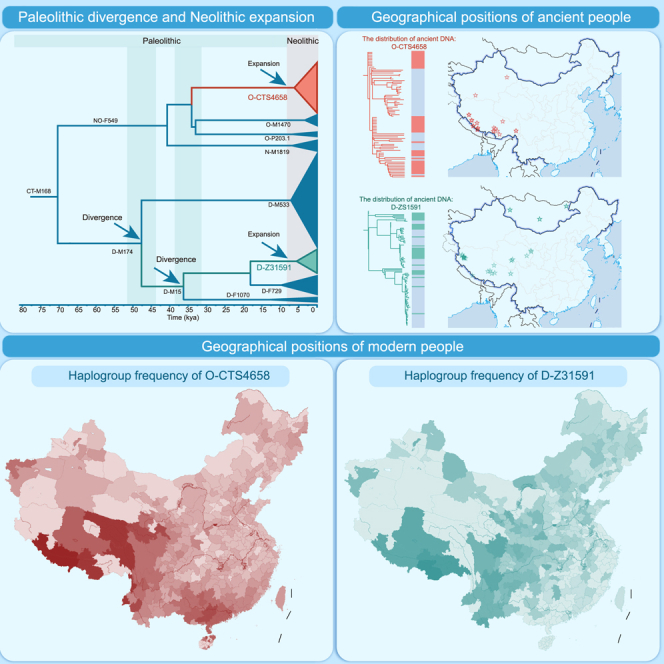

## Introduction

The non-recombining region of the Y chromosome (NRY), which is uniquely inherited along male lines, offers significant potential for applications in forensic science and molecular anthropology. Analyses of the genetic structure and genomic diversity of ethno-linguistically different human populations, informed by databases such as the 1000 Genomes Project, 10K Chinese People Genomic Diversity Project, and the Human Genome Diversity Project, revealed that populations with diverse ethnolinguistic backgrounds possess distinct genetic architectures influencing human traits and diseases.^1^^,^^2^^,^^3^ Initiatives such as the All of Us Research Program enhance the understanding of human genetic diversity by focusing on previously underrepresented populations, thus reducing European bias in genetic research.^4^^,^^5^^,^^6^ Despite abundant genomic resources for mitochondrial DNA and autosomes, comprehensive resources for the Y chromosome remain scarce.^4^^,^^7^^,^^8^^,^^9^^,^^10^ The complexity and high repetitiveness of the Y chromosome sequence have historically hindered detailed studies of its structural variations and the biological implications of its variants. However, recent advancements in capture sequencing and long-read sequencing technologies have facilitated more precise Y chromosome assembly, which is critical for various applications.^11^^,^^12^ The completion of a telomere-to-telomere (T2T) assembly of the Y chromosome, coupled with population genetic analyses of 43 diverse human Y chromosomes, underscores the complexity and variability of their sequencing characteristics and population-specific variations.^11^^,^^12^ These developments significantly advanced the ability to engage high-confidence NRY regions and measurable Y chromosome segments in forensic investigations, population genetic studies, and molecular anthropology, promising substantial impacts on multiple disciplines.

China’s vast genetic, cultural, and ethnic diversity reflects a history shaped by complex movements and admixture events involving ancient Yellow River millet farmers, Yangtze River rice cultivators, diverse Paleolithic hunter-gatherers, and Western Eurasian pastoralists.^13^ This intricate genetic history underpins the spatiotemporal diversity observed in ancient and modern East Asian populations.^8^^,^^13^ The origins and dispersal of the Sino-Tibetan (ST) language family, which is predominant in eastern Eurasia and comprises the Tibeto-Burman (TB) and Sinitic languages, remain debated. Hypotheses suggest that the ST languages originated in North China, the Tibetan-Yi Corridor (TYC) in western Sichuan, and northeastern India on the southern Qinghai-Xizang Plateau.^14^ Analyses of ancient DNA from the Yellow River Basin revealed connections between Neolithic millet farmers and early highland East Asians, including populations in the Qinghai-Xizang Plateau and Nepal.^15^^,^^16^^,^^17^ Mitochondrial DNA and Y chromosomal data have highlighted the Paleolithic origins of the region’s initial settlers and their links to broader East Asian maternal and paternal lineages.^15^^,^^18^^,^^19^^,^^20^ Population genetic studies suggest that the genetic composition of modern Tibetans was shaped by both Paleolithic colonization and Neolithic expansion events.^21^^,^^22^ This is further corroborated by recent ancient DNA studies identifying a Holocene link between millet farmers and ancient Qinghai-Xizang Plateau populations, as well as a deep genetic connection between Tibetans and early Asians.^16^ Due to their varying natural environments and interactions with culturally diverse groups, geographically distinct TB-speaking populations show differentiated population structures.^16^^,^^23^ While core Tibetan populations on the Plateau display unique genetic profiles, those in the surrounding lowlands have been influenced by gene flows from neighboring Indians, Central Asians, and other East Asian populations.^16^^,^^23^^,^^24^ This complex genetic legacy underscores the need for further exploration into paternal genetic diversity and population evolutionary processes among geographically distinct TB groups, offering profound insights into the demographic processes that have shaped regional human history.

Recent Chinese genomic cohorts, such as STROMICS, the China Kadoorie Biobank, ChinaMAP, the NyuWa Genome Resource, and the Born in Guangzhou Cohort Study, have documented the genomic diversity of the Chinese populations.^3^^,^^13^^,^^25^^,^^26^^,^^27^^,^^28^^,^^29^ These studies have significantly contributed to filling the gaps in the genomic data of Chinese populations and advancing human health equity.^5^ Despite these advancements, the genomic resources of the Y chromosome and their potential to elucidate the paternal genetic history of this group have not yet been explored. To address the missing diversity of Y chromosomes in China, we launched the YanHuang cohort, aimed at sequencing over 100K ethnolinguistically diverse Chinese males to delineate the complete genetic landscape of Y chromosome variations and investigate the paternal origins of ancient and modern Chinese populations. Our pilot work reported the paternal genetic background of diverse admixture models within the Han Chinese and ethnic minority groups.^13^^,^^30^ Wang et al. constructed a phylogenetic tree from modern and ancient East Asian populations, revealing multiple founding lineages from ancient farmers, herders, and hunter-gatherers that shaped the paternal gene pool of contemporary East Asians.^13^ Another study highlighted the diverse contributions to East Asian paternal lineages and introduced the "Weakly-Differentiated Multi-source Admixture model" to decode the complex demographic history of Han Chinese populations using extensive genomic data.^30^ However, paternal genomic diversity, settlements on the Qinghai-Xizang Plateau, and potential geographical corridors facilitating population exchange between highland and lowland areas remain uncharacterized in the current era of sequencing.

Y chromosome markers are pivotal in reconstructing paternal demographic history, enhancing forensic paternal biogeographic inferences, and refining pedigree searches.^7^^,^^9^^,^^23^ Specifically, Y chromosome short tandem repeats (Y-STRs) are frequently utilized in genetic research due to their effectiveness.^31^^,^^32^^,^^33^^,^^34^ Analyzing numerous Y-STRs enhances haplotype identification resolution within populations, improving the discriminative capacity of genetic analysis. However, the high mutation rates of Y-STRs, ranging from 1.0 × 10^−4^ to 1.0 × 10^−3^ per generation, introduce challenges by potentially altering haplotypes within the same lineage, complicating forensic familial searches.^35^ Conversely, Y chromosome single nucleotide polymorphisms (Y-SNPs), which have lower mutation rates of approximately 1.0 × 10^−8^ per generation, provide a stable method for preserving paternal lineage information over extensive periods.^36^ Here, we reported large-scale paternal genomic data aimed to refine paternal lineage investigations by distinguishing lineages via the shared haplotypes or haplogroups, thereby providing a clearer picture of the genetic structure and forensic characteristics of geographically distinct TB-speaking populations. This comprehensive approach enhances the understanding of genetic diversity and supports forensic applications by providing more accurate lineage information.

## Results

### YanHuang cohort genomic resources and episodes of Paleolithic and Neolithic diversification and isolation in Tibeto–Burman people

**Figure 1:**
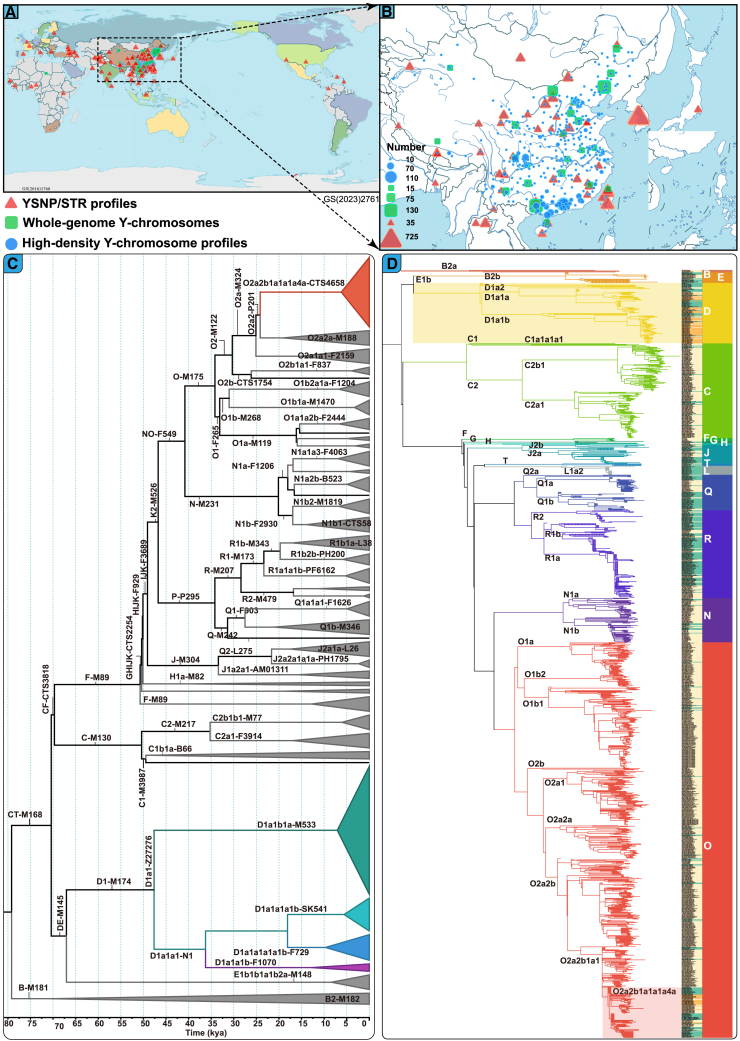
Geographical position, phylogeny, and phylogenetic relationships between modern and ancient populations (A) Geographical distribution of the newly whole-genome sequenced and genotyped Tibeto-Burman (TB)-speaking populations and reference groups. (B) Detailed map of the Chinese regions encompassing the newly collected TB groups. (C) Time-calibrated TB-dominant D and O lineage phylogeny showing the main founding lineage highlighted in this work. (D) Maximum likelihood-based phylogenetic relationships showing a clustering pattern between modern and spatiotemporally different ancient populations.

We present an integrated YanHuang Y chromosome genomic resource encompassing data from 9,901 ethnolinguistically diverse individuals across 38 ethnic groups and 34 provinces (Figures 1A and 1B). This objective was to identify the founding lineages of TB people and reconstruct their paternal demographic history. The dataset comprises three distinct types of data. First, whole Y chromosome sequences from 994 modern and 303 ancient individuals^16^^,^^18^^,^^37^^,^^38^^,^^39^^,^^40^^,^^41^ were used to reconstruct the phylogenetic relationships between modern and ancient Chinese populations and estimate the chronology of divergence, expansion, and migration events in modern and ancient TB populations. Second, we analyzed 4,298 genetic profiles featuring population-specific SNP and STR variations from Chinese populations to explore the genetic relationships and landscape of TB people and other reference Chinese populations.^42^^,^^43^^,^^44^ Finally, we examined genetic data from 4,306 individuals with high-density Y-SNPs from 38 ethnic groups in the Chinese Paternal Genomic Diversity Project (CPGDP) to elucidate the origins and dispersal patterns of two TB-related founding lineages.

Y chromosome sequences provide insights into the common patrilineal ancestors of founding lineages. We sequenced genomes from 72 TB-speaking representative samples and integrated them with modern and ancient Eurasian data from the pilot work of the YanHuang cohort,^13^ creating a comprehensive dataset of 1,297 Y chromosomes. The four B2b1a1b African lineages served as the basal branch in our time-stamped phylogenetic analysis. This analysis revealed a coalescence between the D and O founding lineages 65,339 to 74,810 years ago (ya). Divergence and admixture events, indicated by BEAST analyses, suggested prolonged population bottlenecks followed by recent expansions in these lineages. Specifically, the D1a1a and D1a1b lineages diverged between 43,354 and 51,057 ya after a 19,270-year bottleneck. The D1a1a lineage then split into the D1a1a1b and D1a1a1a1b sublineages after an 11,340-year period of stability, after which it expanded during the Neolithic transition (Figure 1C). We identified two Neolithic TB-related lineages of the O2a2b1a1a1a4a-CTS4658 and D1a1a1a1b-Z31591 expanded in TB people. The Tibetan-dominant D1a1a1a1b lineage expanded between 4,692 and 6,663 ya, likely coinciding with the Proto-Tibetan adoption of millet or barley farming and adaptation to high-altitude environments. Similarly, a lineage associated with Sherpa and other Tibetan populations expanded between 5,403 and 7,040 ya, as observed in the Pumi and other groups.

**Figure 2:**
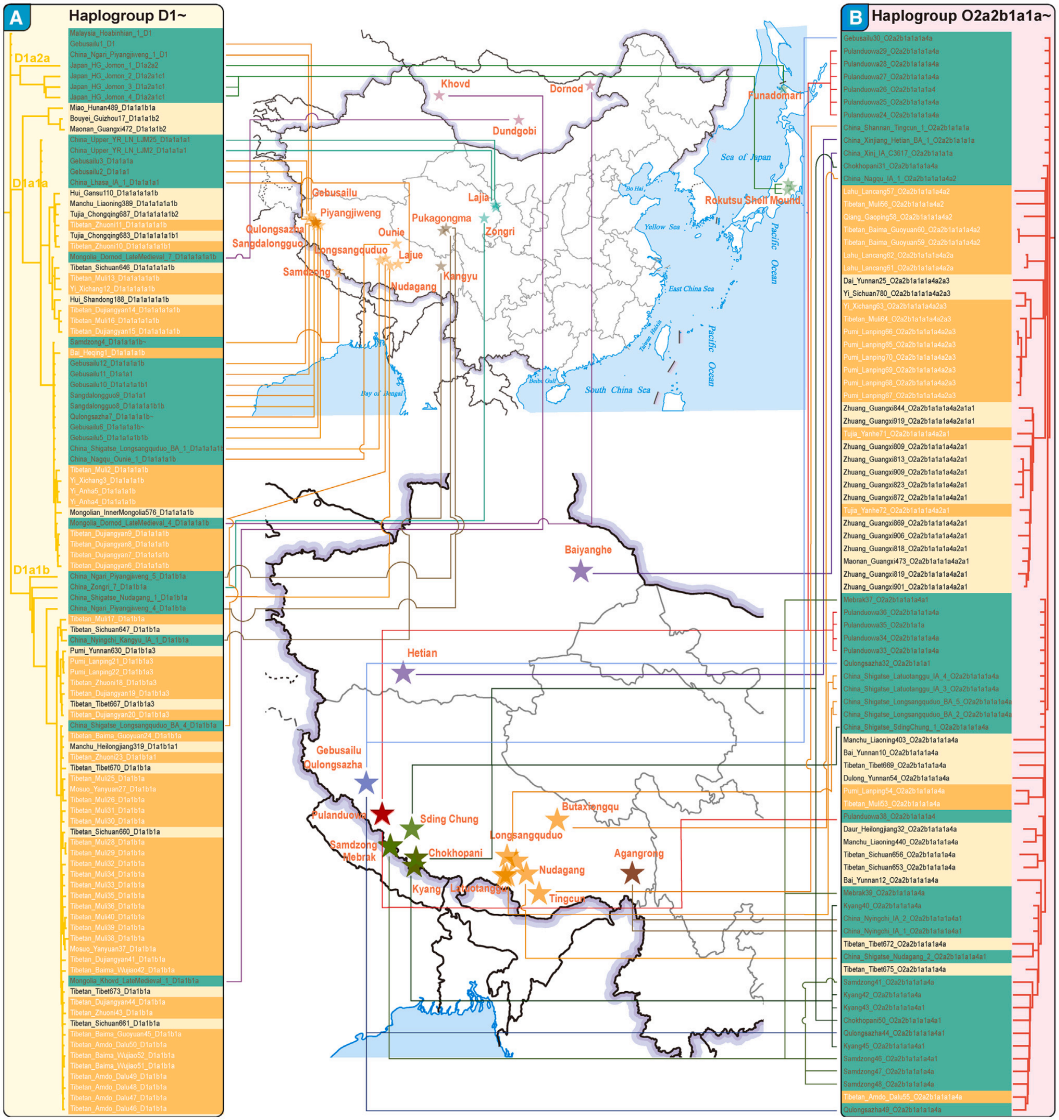
Geographical position and pathPhynder placement of ancient East Asian samples belonging to two TB-founding lineages into this fully resolved Y chromosome phylogeny (A) Phylogenetic and clustering patterns of D lineages among modern TB people and ancient eastern Eurasian individuals. (B) O lineages carried by modern TB people, Tai-Kadai people, and ancient highland Qinghai-Xizang Plateau individuals. The geographical positions of key ancient individuals were labeled in the middle maps. Ancient individuals were denoted via the green background. The base map was officially approved with the number GS(2019)1674 (http://bzdt.ch.mnr.gov.cn/).

Further exploration of phylogenetic patterns among modern and ancient populations led to the construction of a unified paternal genealogy (Figure 1D). The early population structure, associated with multiple typical East Asian lineages, revealed that at least two distinct ancestral founding lineages contributed to the genetic pool of the TB-speaking populations. Phylogenetic analyses among modern and ancient East Asians confirmed genetic continuity during the Neolithic period across major genetically differentiated regions: the northern Yellow River Basin, southern Yangtze River Basin, Amur River Basin, and Qinghai-Xizang Plateau. The D1a2 lineages found in the Jomon people represent an early divergence from the Qinghai-Xizang Plateau-related D1a1 lineages. Two primary sublineages of D1a1, D1a1a, and D1a1b diverged during the Upper Paleolithic period. The D1a1a lineage was identified in both modern Tibetan and Yi populations, as well as in ancient individuals from the Qinghai-Xizang Plateau (Figure 2A), including those from archeological sites such as Samdzong, Gebusailu, Qulongsazha, Sangdalongguo, and Gebusailu. The D1a1b lineage was observed in the Tibetan and Yi populations, as well as in the Mosuo and Pumi populations and in Iron Age individuals from Nyingchi Kangyu (D1a1b1a). The O2a2b1a1a1a4a lineages were identified in the Yi, Lahu, Pumi, and previously documented Zhuang populations, which clustered with 39 individuals from the Bronze Age to historical periods in highland areas (Figure 2B). Meanwhile, the N1b2 lineage has been observed in modern Yi and Tujia populations, as well as in several ancient individuals from the Qinghai-Xizang Plateau (Sangdalongguo, Laga, Gebusailu, Qulongsazha, and Zongri). Additionally, four ancient individuals from high-altitude regions belong to the O2a2b1a2a1a lineage. Overall, TB-related O-CTS4658 and D-Z31591 lineages were identified in both modern and ancient TB individuals, which clustered with a diverse range of ancient highland populations.

### Genetic diversity of Tibeto–Burman people inferred from Y-STR haplotypes

**Figure 3:**
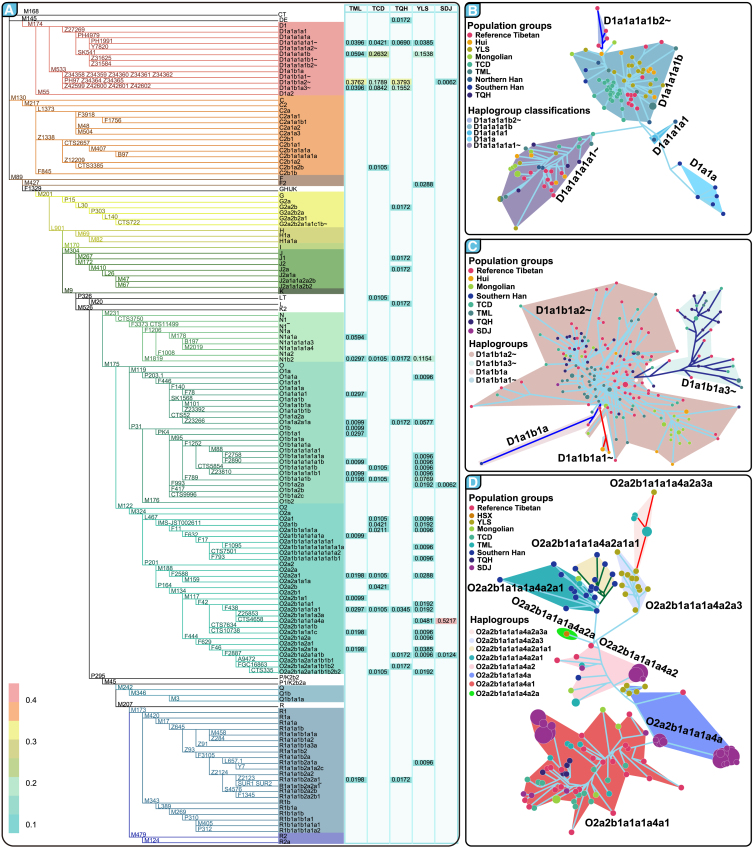
Fully-resolved Y chromosome phylogeny and paternal genetic history of TB people (A) High-resolution phylogenetic tree and haplogroup frequency heatmap for five TB-speaking populations. This figure presents a streamlined phylogenetic tree alongside a heatmap that illustrates the distribution frequencies of various haplogroups across five distinct TB-speaking populations. (B) Median-joining network topologies derived from Y-SNP-STR haplotypes. This series of networks elucidates the genetic relationships and evolutionary divergence within key paternal lineages among the studied populations, with each panel focusing on different levels of haplogroup resolution. Network topology for D1a1a-M15 subhaplogroups, which were denoted via different backgrounds. Network topology illustrates the diversity within D1a1a-M15 subclades, which is denoted by the different colors of the circle. (C) Network depicting the structure of the D1a1b-P99 subhaplogroups. Detailed topology of D1a1b-P99 subhaplogroups, highlighting specific lineage relationships. (D) Network topology for O2a2b1a1a1a4a-CTS4658 sublineages showing branching patterns. Detailed view of the haplogroup distribution within the O2a2b1a1a1a4a-CTS4658 sublineages.

**Figure 4:**
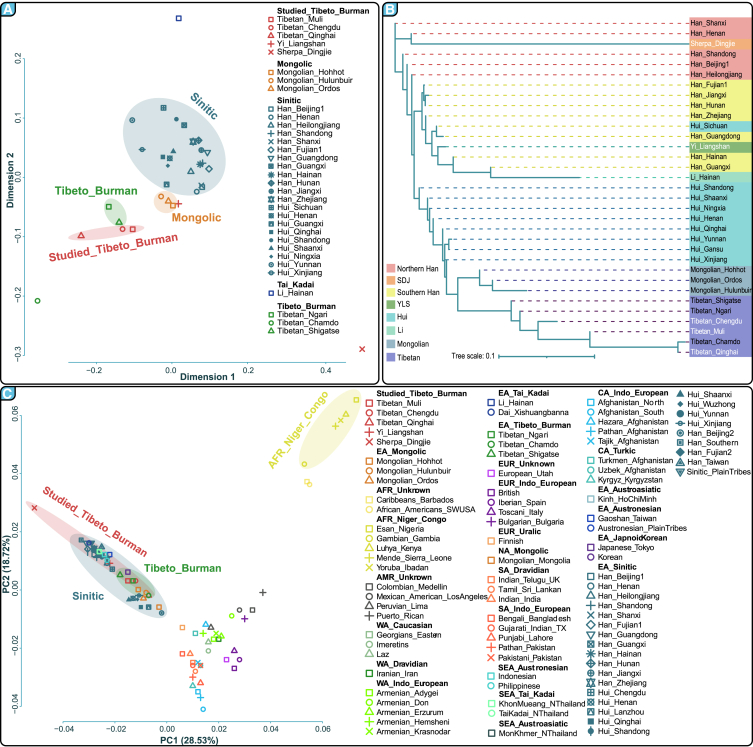
Geographical distribution and genetic relationships of newly collected and reference populations (A) Multidimensional scaling analysis based on the Fst genetic distance matrix comparing newly collected TB populations with 28 Chinese reference groups. (B) Neighbor-joining phylogenetic tree derived from the 27-Y-STR-based Rst genetic distance matrix illustrating the genetic relationships among the populations studied. (C) Principal component analysis depicting clustering patterns among target TB-speaking populations and 87 global reference populations. The base map was officially approved with the numbers GS(2016)1760 and GS(2023)2761 (http://bzdt.ch.mnr.gov.cn/).

To comprehensively explore the genetic patterns of the TB-speaking population and their relationships with other reference groups, we genotyped paternal diversity data of large-scale populations via more cost-effective genotyping methods. We reported 4,298 Y chromosome haplotypes, including 37 Y-STRs and 215 Y-SNPs, with 519 newly genotyped TB individuals. These data were submitted to the YHRD database, revealing significant genetic diversity across ethnolinguistically distinct populations (Figures 3 and 4). Among the TB-speaking individuals, 495 unique Y-STR haplotypes were identified, distributed as follows: 85 Tibetans in Muli (TML), 93 Tibetans in Chengdu (TCD), 104 Yis in Liangshan (YLS), 137 Sherpas in Dingjie (SDJ), and 58 Tibetans in Qinghai (TQH), indicating considerable genetic heterogeneity. The shared haplotype between the two populations highlighted their genetic interconnectedness. The haplotype diversity (HD) ranged from 0.9978 to 1.0000, demonstrating the robust discrimination power of our genetic profiling, especially when using the AGCU Y37 kit (Table S5). This kit outperformed Yfiler Plus and Yfiler in delineating genetic diversity due to its overall higher discrimination capacity (DC) and lower haplotype match probability (HMP) in the studied population (Table S5), supported by the analysis of 234 alleles across 31 single-copy loci. (Table S6). We identified a unique 20.3 microvariant allele at DYS627, traced to a 'G' deletion at the 19th repeat unit via Sanger sequencing (Figure S1). The analysis of three multicopy loci—DYS527, DYS385, and DYF387S1—revealed 133 allele combinations, indicating significant genetic diversity, with rapidly mutating Y-STRs (RM Y-STRs) showing greater diversity than slower-mutating loci such as DYS391, DYS437, and DYS645 (Tables S6 and S7). Comprehensive Y-SNP-STR analysis associated all samples with microvariant alleles at DYS518 and the Q1a1-F746 haplogroup, enhancing our understanding of genetic structures and refining paternal lineage analysis for forensic and anthropological applications (Table S8).

Our findings revealed an increase in shared haplotypes and a decrease in discrimination capacity as the number of genotyping markers decreased. This underscores the need for a tailored Y-STR panel for Chinese populations to reduce the risk of false matches in forensic applications. Genetic diversity (GD) assessments indicated that multicopy loci exhibited the highest diversity, with RM Y-STRs showing significant diversity. However, due to their high mutation rates, RM Y-STRs are less suited for paternal kinship identification, whereas conventional Y-STRs, which mutate more slowly, are preferred for reliable lineage tracing. Our integrated analysis linked all samples with microvariant alleles at DYS518 to the Q1a1-F746 haplogroup. This finding emphasizes the necessity of considering both allele and haplogroup data to clarify genetic lineage and historical migratory events, thereby enhancing the accuracy and reliability of forensic and genealogical investigations.

### Y chromosome haplogroup distribution among geographically diverse Tibeto–Burman groups

We identified a wide range of Y chromosome haplogroups across geographically distinct TB-speaking populations. Specifically, 44 haplogroups were observed in 254 Tibetan individuals: 25 in TML, 19 in TCD, and 19 in TQH. For YLS, 33 haplogroups were identified, while SDJ exhibited only seven, demonstrating reduced genetic diversity (Figure 3A). The Haplogroup diversity (HGD) varied significantly, from a low of 0.6183 in SDJ to a high of 0.9376 in YLS. Prevalent haplogroups among Tibetans included D1∗-M174, which was found in more than half of the individuals across the three Tibetan subpopulations, and O2∗-M122, which was notably more frequent among YLS individuals. Additionally, O1b∗-P31, N∗-M231, and O1a∗-M119 were common in YLS, with N1b2-M1819 being the dominant subhaplogroup of N-M231. In the SDJ, O2∗-M122 dominated (98.14%). Subhaplogroup analysis revealed distinct distribution patterns. For example, in Tibetan populations, subhaplogroups D1-M174, such as D1a1a1a1b-SK541 and D1a1b1a2∼-PH97/Z34364/Z34365, showed variable frequencies across regions, with D1a1a1a1b-SK541 being the most frequent in YLS. In contrast, the subclade O2a2b1a1∗-M117 of O2-M122 was most prevalent across the Tibetan and Yi populations, highlighting different genetic legacies and geographical differences. This detailed haplogroup profiling underscores the complex genetic landscape of TB-speaking populations and provides crucial insights into their historical migrations and interactions. The distinct haplogroup compositions reflect the unique evolutionary histories and adaptive strategies of these populations at different altitudes, influenced by both their environment and their historical migration patterns.

We extensively investigated the phylogeographic distribution of founding haplogroups among TB speakers. Our findings indicated the prominent presence of the D1a1a haplogroup in Southwest China, particularly among TB-speaking communities (Figure S2A). The D1a1b haplogroup showed a broader regional presence in both Southwest and Northwest China, especially among geographically diverse Tibetan groups (Figure S2B). The O2a1 haplogroup, predominantly found among East Asians (including many Han Chinese individuals), was notably absent in Northwest East Asians (Figure S2C). Conversely, O2a2 exhibited a broad distribution across East and Southeast Asians (Figure S2D). The N1a haplogroup was primarily found in northern European and northern East Asian populations, while N1b appeared predominantly in southwestern East Asia (Figure S2E and S2F). Additional haplogroups, such as C2∗-M217, G∗-M201, and J∗-M304, were present in minor frequencies within these populations, underscoring a complex mosaic of paternal lineages in geographically diverse regions of China (Figure 3A). Our data also revealed rich diversity within Tibetan populations, including rare haplogroups such as LT-P326 and L-M20 (Figure 3A), suggesting varied historical interactions and migrations. This analysis provides a comprehensive overview of the genetic structure within TB-speaking populations, highlighting the significant variation and widespread distribution of specific haplogroups and enhancing our understanding of their historical and evolutionary backgrounds.

### Fine-scale paternal genetic structure of Tibeto–Burman people

#### Genetic similarities and differences revealed by Y-STR haplotypes

Population genetic work suggested that STR markers with high mutation rates have a stronger power to illuminate recent dynamics of human genetic history.^45^ We analyzed Y-STR haplotype data to investigate the paternal genetic structure of TB speakers, revealing significant genetic relationships within and across TB populations (Figures 4A‒4C, S3‒S5, and Tables S9, S10, and S11). The initial findings showed significant genetic proximity within regional subgroups (Figure S3; Table S9). For instance, the TML and Tibetan in Nagqu (TNG) populations exhibited no measurable genetic distance, indicating strong genetic continuity. Similarly, Tibetan subpopulations such as Tibetan in Shigatse (TSG) demonstrated close genetic affiliations with TML, suggesting regional genetic coherence among highland Tibetan communities. Using 29 Y-STR markers, we confirmed that Tibetan populations share more genetic similarities with other highland groups than with lowland East Asian populations (Figure S4; Table S10). The YLS population showed a close genetic affinity with the Qiang in Beichuan (QBC) population, underscoring shared genetic traits across geographically and culturally connected groups. In contrast, the SDJ population was markedly distinct from other TB-speaking groups, aligning more closely with certain lowland Han populations. This highlights the complex mosaic of genetic influences in this region due to historical migrations and interactions. These results underscore the importance of regional and cultural contexts in shaping genetic structures, contributing to a deeper understanding of genetic diversity within East Asian populations.

The analysis of 27 Y-STR haplotypes (Figure S5; Table S11) confirmed close genetic relationships among Tibetan groups, highlighting significant affinities, particularly between the YLS and Sichuan Hui populations. The SDJ population showed closer genetic ties with the Han populations of Henan and Shanxi, with Rst values of 0.2634 and 0.2663, respectively. MDS based on 29 Y-STRs revealed a distinct Tibetan-related cluster, underscoring strong genetic links across Tibetan populations in different geographic locations (Figure S6; Table S10). This is notable among Tibetan groups in the TYC and Qinghai-Xizang Tibetan, and between geographically different Tibetan groups in Northwest China. Interestingly, the SDJ population appeared to be genetically isolated from other East Asian groups, corroborated by high Rst genetic distances greater than 0.21 (Table S10). Additionally, MDS analysis using 29 Y-STRs indicated that YLS shows greater genetic similarity with the linguistically related Yi population in Guizhou than with the geographically close QBC population. Conversely, the 27-Y-STR-based MDS revealed a cluster predominantly associated with Sinitic languages, positioning the SDJ and Hainan Li populations as distinct from the other analyzed groups (Figure S7). In this context, the TML closely aligns with other highland Tibetans, while the TCD and TQH are distinct from typical high-altitude Tibetan populations. The clustering of the YLS and Hui populations from Shaanxi underscores their shared genetic makeup, emphasizing the complex interplay of geography, language, and genetics in shaping the population structure of East Asia.

#### Clustering patterns revealed by Y-SNP haplotypes and haplogroups

Y-SNP haplotype analysis revealed clear clustering patterns among Tibetan and other East Asian populations. The TML and TCD populations were closely related to TNG and TSG. Similarly, TQH showed significant genetic links to TSG and TCD. YLS exhibited notable genetic affinity with the Hui population in Xinjiang, reflecting shared paternal lineages and regional genetic influences. The SDJ group shared genetic closeness with the northern Han Chinese, possibly indicating historical migrations or genetic admixture (Tables S12, S13, Figures S8 and S9). MDS analyses of 113 overlapping Y-SNPs suggested that the newly studied Tibetan populations (TML, TCD, and TQH) formed a distinct cluster; YLS was closely related to TSG, while SDJ was distinctly separate from other global populations (Figure S10). Additional MDS analysis using 157 Y-SNPs revealed that TML and TCD grouped with TSG, highlighting strong regional genetic coherence, while YLS aligned more closely with Mongolian reference populations (Figure 4A). Phylogenetic analyses confirmed the genetic proximity of Tibetan populations across different regions and underscored the distinct genetic makeup of the SDJ population (Figures 4B, S11, and S12). This complex genetic landscape illustrates the diverse genetic heritage of TB-speaking populations and underscores the impact of geographic separation and historical migrations on genetic diversity.

PCA patterns based on haplogroup frequencies revealed significant insights into the population structure of TB-speaking groups. We identified distinct clusters associated with geographic and ethnic origins (Figures 4C and S13). An extensive Asian-related gradient stretched from southern Han Chinese to Pathan populations in Afghanistan, while European and American populations aligned along the second principal component (PC2), showing diverse genetic backgrounds (Figure 4C). Within East Asia, a pronounced north‒south genetic gradient encompassed Han-, Hui-, and Mongolian-related clusters. This gradient was particularly marked in a focused analysis of East Asian populations, where Hui/Mongolian and Tibetan-related clusters were distinct, and the Austronesian-speaking Gaoshan population of Taiwan and Han Chinese from Shanxi occupied the extremes (Figure S13). The target Tibetan populations aligned closely with other East Asian groups, reflecting shared regional heritage. Notably, the YLS population showed closer genetic affiliation with the Hui population from Henan, while the SDJ population was isolated from other East Asian groups, indicating complex historical interactions and migrations within these regions.

### Evolutionary history patterns inferred from the combined analysis of Y-SNP-STR haplotypes

To investigate the distribution patterns and evolutionary trajectories of major haplogroups among TB groups, we utilized MJ network topologies constructed from haplotypes derived from 27 Y-STRs and 157 Y-SNPs (Figures 3B‒3D and S14‒S17). We found that haplogroup D1a1a∗-M15, particularly its subhaplogroup D1a1a1a1b-SK541, was mainly present at low and middle altitudes in the TCD and YLS populations, with a lower prevalence among highland Tibetans (Figure 3B). Another significant subhaplogroup, D1a1b∗-P99, especially D1a1b1a2∼-PH97/Z34364/Z34365, was distributed across various Tibetan populations, indicating broad geographical spread among high-altitude communities (Figure 3C). Our analysis revealed that haplogroup O2∗-M122 was significantly prevalent across TB-speaking populations. Within this group, subhaplogroup O2a1∗-L467 was widespread among Han Chinese individuals, whereas O2a2a∗-M188 was predominant in southern Han Chinese individuals (Figures S14 and S15). Subhaplogroup O2a2b1a1∗-M117 exhibited high frequencies among the newly studied TB speakers and the Sinitic-speaking Hui and Han populations (Figure S16). O2a2b1a2a∗-F444 was notably prevalent among the Han and Hui populations (Figure S17). Particularly striking was the prominence of O2a2b1a1a1a4a-CTS4658 and its subhaplogroups in the SDJ, where it formed a star-like topology in the MJ network, suggesting a recent rapid expansion in this highland population (Figure 3D). This subhaplogroup also showed significant differences between the Tibetan and Yi groups, although it was less common in the Han Chinese population. These findings enrich our understanding of the complex genetic makeup and historical migrations of TB groups across different regions of Asia.

To investigate factors influencing genetic diversity among ethnolinguistically and geographically distinct populations, we conducted an AMOVA using 27 Y-STR and 157 Y-SNP markers across 33 Chinese populations categorized by ethnicity, linguistic affiliation, and altitude. Our analysis revealed that variations among groups and populations derived from 157 Y-SNPs were significantly greater than those from 27 Y-STRs (Table S14). Specifically, among-group variations based on ethnic categorization (15.32% for 157 Y-SNPs and 5.41% for 27 Y-STRs) exceeded those based on linguistic (8.70% for 157 Y-SNPs and 2.34% for 27 Y-STRs) or altitude groupings (6.65% for 157 Y-SNPs and 2.70% for 27 Y-STRs). Within-group variations among populations sharing the same altitude or linguistic family were notably more pronounced than those between ethnically similar groups. Intrapopulation variations accounted for the majority of genetic differences among Chinese populations, exceeding 82% for 157 Y-SNPs and 93% for 27 Y-STRs. These findings underscore the enhanced discriminatory power of 157 Y-SNPs over 27 Y-STRs and their utility in tracing paternal lineages among diverse Chinese groups.

### Phylogeographical origin of Tibeto–Burman founding lineages

**Figure 5:**
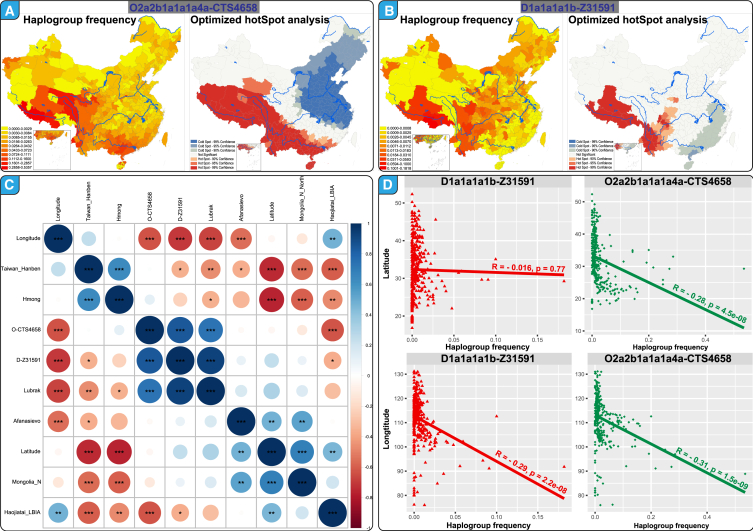
Phylogeographical analysis and correlation results of two TB-related founding lineages (A and B) Haplogroup frequency and optimized hotspot analysis results for O2a2b1a1a1a4a-CTS4658 and D1a1a1a1b-Z31591. The red color in the left panel indicates a higher haplogroup frequency, and the yellow color indicates a low haplogroup frequency in the frequency spectrum. The red color in the right panel denotes the possible original center. (C) The correlation between the founding lineage frequency and the geographical coordinates and ADMIXTURE-based admixture proportion using Pearson correlation analysis. The blue color indicates a positive correlation, and the red color indicates a negative correlation. ∗ represents 0.05 < *p* value <0.01, ∗∗ represents 0.01 ≤ *p* value <0.001, ∗∗∗ represents *p* value <0.001. (D) The correlation between the frequency of two founding lineages and the latitude and longitude coordinates using Pearson correlation analysis. The base map was officially approved with the number GS(2023)2767 (http://bzdt.ch.mnr.gov.cn/).

We finally examined the phylogeographical distribution of key mutations within the CPGDP resource. From a cohort of 232,413 individuals, we screened 918 samples from the D-Z31591 lineage and 3,388 from the O-CTS4658 lineage. Among the D-Z31591 sublineages (D-MF122648, D-F2625, D-F3401, D-F19133, and D-MF175572), we observed substantial population expansions. Notably, we collected 13 samples from Xizang, 8 from Qinghai, and 155 from Sichuan (Figure 1B). Analysis of haplogroup frequency and Y-SNP/STR profiles indicated that the highest frequencies occurred on the Qinghai-Xizang Plateau, suggesting that this region was a potential origin and center of post-colonization expansion for these ancient highlanders (Figure 5A). This pattern was further supported by optimized correlation analysis. Within the D-Z31591 lineage, 793 samples were from Hans, 33 from Tibetans, and 21 from Yis, representing the top three ethnic groups. For O-related TB founders, equivalent methodologies revealed the highest frequencies predominantly in the Qinghai-Xizang Plateau and Southwest China, as supported by optimized hotspot analysis (Figure 5B). Pearson correlation analysis between geographical coordinates and prefecture-level frequencies indicated no significant correlation with latitude for the D-Z31591 lineage and marginal negative correlations for other parameters (Figure 5D). These results suggest that the formation of these lineages in highland and lowland East Asians was not solely driven by isolation by distance. Finally, we analyzed the correlation between human migration patterns inferred from autosomal and Y chromosome evidence by comparing ADMIXTURE-based ancestral proportions and lineage frequencies. A positive correlation emerged between our identified lineages and Lubrak-related highland East Asian ancestry (Figure 5C), underscoring a significant genetic link.

## Discussion

Previous genetic studies on paternal genetic diversity have sought to elucidate the formation of East Asia through preglacial and postglacial migrations via southern and northern routes.^46^^,^^47^^,^^48^^,^^49^ These studies also examined complex migrations and admixture within and between lowland and highland East Asia using low-density Y-SNP variations^22^^,^^31^^,^^32^^,^^50^^,^^51^ and sex-biased adaptations shaping uniparental gene pools.^47^ The Qinghai-Xizang Plateau, known for its harsh environmental conditions such as high altitude, low temperatures, severe aridity, and oxygen scarcity, has been home to human settlement since the Paleolithic era.^49^^,^^52^^,^^53^ Despite these formidable challenges, modern humans established themselves in the region, with many Paleolithic sites across the plateau dating back to around 20,000 ya.^49^^,^^54^ However, genetic research reveals that present-day Tibetan populations have their origins in Neolithic East Asia, specifically northern China.^14^^,^^55^ Recent gene flow has been strongly indicated by previous studies.^49^^,^^56^ However, fine-scale paternal genetic history from eastern regions, including the northeastern Qinghai-Xizang Plateau and the TYC, remains largely unknown, particularly from large-scale high-density Y-SNP data or whole-genome sequencing data. We reported an integrated YanHuang Y chromosome genomic resource, focusing on the formation of modern highland East Asians through whole Y chromosome sequencing, Y-SNP/STR genotyping of TB-speaking individuals, and high-density Y-SNP data from ethnolinguistically diverse Chinese populations across 34 provinces. Our study identified prevalent paternal lineages within highland TB-speaking populations, highlighting haplogroups D1∗-M174 (including subhaplogroups D1a1a1a1b-SK541, D1a1b1a2∼-PH97/Z34364/Z34365, and D1a1b1a3∼-Z42599/Z42600/Z42601/Z42602) and O2∗-M122, especially O2a2b1a1∗-M117. The TB-speaking SDJ predominantly exhibited haplogroup O2a2b1a1a1a4a∗-CTS4658, indicating a unique paternal lineage. Among the lowland TB-speaking YLS, the dominant haplogroup was O2∗-M122 (O2a2b1a1∗-M117 and O2a2b1a2a∗-F444), with significant occurrences of D1∗-M174 (D1a1a1a1b-SK541), N∗-M231 (N1b2-M1819), O1b∗-P31 (O1b1a1∗-PK4), and O1a∗-M119 (O1a1a∗-P203.1), indicating diverse genetic backgrounds. The D1-M174 haplogroup, integral to the East Asian paternal lineage, is particularly frequent among Tibetan and some Japanese populations, illustrating its historical significance and geographical spread.^57^ Variations within this haplogroup, such as D1a1a∗-M15 and D1a1b∗-P99, underscore their regional importance^22^ and are often considered Tibetan-specific lineages. The presence of this lineage across different Tibetan groups from Muli to Qinghai suggests a deep-rooted and widespread historical influence. It is widely believed that haplogroup D-M174 represents the remnants of the earliest modern human settlers on the Qinghai-Xizang Plateau, who likely endured through the Last Glacial Maximum.^22^^,^^49^ The migration patterns of D1a1a-M15, derived from D1-M174, highlight its evolution and expansion from western Sichuan northward into Qinghai and across the TYC into the Himalayas, reflecting significant migratory events and adaptations.^58^ These genetic insights enrich our understanding of the paternal genetic structure among Tibetan-speaking populations and enhance our knowledge of their historical migrations and interactions across diverse ecological and geographical landscapes.

The haplogroup O2∗-M122, predominant among the newly analyzed Tibetan-speaking populations, is widely distributed across East and Southeast Asia.^32^^,^^42^^,^^59^^,^^60^^,^^61^ Studies, including those by Yan et al., indicate that approximately 40% of Han Chinese people trace their paternal lineage to late Neolithic progenitors, particularly from the Oα (O2a2b1a1∗-M117), Oβ (O2a2b1a2a1a∗-F46), and Oγ (O2a1b1a1a1a∗-F11) lineages.^60^ These lineages significantly shaped the paternal genetic landscape of East Asian populations during the Neolithic period. Previous studies have confirmed that approximately 6,000 ya, farmers from the Yangshao culture in the middle Yellow River basin, carrying the O2a2b1a1a-F5 lineage, migrated to the Qinghai-Xizang Plateau.^49^^,^^62^ Additionally, based on ancient DNA from the Banpo site, it is possible that the Yangshao culture also contributed to the spread of haplogroup O2a1b1a1a1a-F11.^63^ The subhaplogroup O2a2b1a1a1a4a∗-CTS4658 was notably prevalent among the Sherpa population, with network analyses indicating recent rapid expansion. This high frequency in the SDJ population may be due to the localized population of Sherpas in China, primarily residing in Dingjie County within the Tibet Autonomous Region, highlighting the genetic distinctiveness of the Sherpa and Tibetan communities on the Qinghai-Xizang Plateau. Meantime, the phylogeographic analysis confirmed the highest frequency among highland Tibetans and their neighbors. Taken together, our time-labeled phylogeny of the O and D lineages, along with phylogenetic relationships among modern and ancient Chinese populations, confirmed that both Paleolithic and Neolithic genetic legacies contributed to the formation of proto-TB populations.

The haplogroup O1a1a∗-P203.1, with its upstream haplogroup O1a-M119 observed in the remains from the Liangzhu site,^64^ predominantly observed in the YLS, is widespread among southern Chinese and Southeast Asian populations and appears among eastern and northern Han Chinese populations.^42^^,^^65^^,^^66^ Subhaplogroups of O1b∗-P31, notably O1b1a1∗-PK4, frequently found in the TML and YLS, are prevalent across southern Chinese and South Asian populations, Southeast Asian tribal communities, and even among the Japanese population.^42^^,^^66^^,^^67^^,^^68^ Additionally, ancient DNA sequences confirm that around 3,000 ya, the Wucheng people in Jiangxi Province carried the O1b1a1a-M95 lineage.^64^ Conversely, the sublineage O1b2∗-M176 is common in Japanese, Korean, and some Manchu populations.^57^^,^^67^ The strategic positioning of the TML and YLS along the TYC, a significant migratory route to the Qinghai-Xizang Plateau, highlights the influence of ancient southern East Asian migrations carrying O1b-related subhaplogroups on the genetic landscape of modern ST-speaking populations,^69^^,^^70^ explaining the relatively high frequency of O1b∗-P31 observed in the TYC populations.

To elucidate the genetic relationships and differences among geographically different TB groups and various East Asian reference populations, we conducted genetic analyses, including genetic distance estimations, MDS, PCA, AMOVA, and phylogenetic relationship construction. These analyses utilized data from haplogroup frequencies, Y-STR/Y-SNP haplotypes, high-density SNP profiles and whole-genome sequences. Notably, the results based on Y-SNP haplotypes and haplogroup frequencies provided a more precise reflection of genetic affinity and differentiation among the ethnolinguistically diverse groups compared to Y-STR haplotypes. This enhanced resolution underscores the value of using diverse genetic markers to capture the complex patterns of genetic affinity and differentiation within and between populations.

Our analysis aimed to enhance the understanding of paternal demographic history among diverse TB-speaking populations. We found that the TML and TCD populations maintained close genetic ties with the Ü-Tsang Tibetans, notably the TSG and TNG groups. In contrast, the TQH population was more genetically aligned with the Kham Tibetan population, particularly the Tibetan_Chamdo population from the eastern Qinghai-Xizang Plateau. The YLS population showed a significant genetic affinity with Hui populations from Sichuan, Shaanxi, and Henan, suggesting considerable gene flow from these regions into the Yi population in the TYC. Conversely, the Sherpa population exhibited distinct genetic traits, supported by unique haplogroup distributions observed in the SDJ, indicating their relative genetic isolation from other groups.

### Limitations of the study

This study has certain limitations, such as limited sampling locations and high coverage of Y chromosome variations. Tibetan populations are widely distributed across the Qinghai-Xizang Plateau, Qinghai, and Sichuan, with smaller populations in Gansu and Yunnan. Expanding sampling to these regions would help provide a more comprehensive understanding of the population history of TB groups. Additionally, much of the data used in this study relies on genotyping and haplogroup frequency information. In the era of whole-genome sequencing, using whole Y chromosome sequences could capture more genetic information and offer deeper insights. Lastly, incorporating large-scale ancient DNA data from the Paleolithic and Neolithic periods in South Asia, surrounding areas of the Qinghai-Xizang Plateau, and the Yellow River basin and Yangtze River basin would further elucidate the complex and dynamic genetic landscape of Tibeto-Burman populations.

### Conclusion

This study utilized three kinds of advanced Y-SNP genotyping technologies to create a valuable genetic resource for forensic genetics and molecular anthropology. Our analysis highlights a strong correlation between specific allelic variations in Y-STRs and well-defined haplogroups, providing a theoretical framework for predicting haplogroups from Y-STR haplotypes. Despite variability within Y-STR haplotypes across similar haplogroups in Chinese populations, we found a consistent association of identical Y-STR haplotypes with specific haplogroups. This confirms a robust relationship between Y-STR haplotypes and haplogroup classifications. This study also revealed a distinct correlation between the complex paternal genetic structures of Chinese populations and their geographical and linguistic contexts. This finding underscores the utility of Y chromosomal markers in forensic pedigree analysis and paternal biogeographical ancestry assessments. Our findings suggest that geographically diverse TB groups exhibit distinct paternal genetic histories yet share close genetic ties with northern lowland East Asians, supporting a shared origin in North China for the ST people. Overall, this work deepens our understanding of genetic diversity and underscores the broader applicability of genetic markers in anthropological and forensic investigations.

## Resource availability

### Lead contact

Further information and requests for genomic resources should be directed to the lead contact, Guanglin He (guanglinhescu@163.com).

### Materials availability

This study did not generate new unique reagents.

### Data and code availability

• Data: The Y-STR and Y-SNP haplotype data for 519 TB-speaking individuals have been deposited in the YHRD database (https://yhrd.org/) under accession numbers YA004726 (TCD), YA004729 (TML), YA004613 (TQH), YA004223 (YLS), and YA004730 (SDJ). The supplementary materials contain all additional data used in this study. The data collection and usage adhered to the guidelines stipulated by the People’s Republic of China on the administration of human genetic resources.
• Code: This article does not report the original code.
• All other items: Requests for access to the raw data should be directed to Guanglin He at guanglinhescu@163.com or Mengge Wang at Menggewang2021@163.com.

## Acknowledgments

We express our gratitude to all the volunteers who contributed to this study. We acknowledge the financial support received from the National Natural Science Foundation of China (Grant No. 82202078) for M.W., (Grant No. 82402203) for G.H., and from the National Social Science Foundation of China (Major Project Grant No. 23&ZD203) for G.H., and from Open Research Project of the Ministry of Public Security (Grant No. 2024FGKFKT02) for M.W. Additional support for G.H. includes the Open Project of the Key Laboratory of Forensic Genetics of the Ministry of Public Security (2022FGKFKT05), the Center for Archaeological Science of Sichuan University (23SASA01), the 1‧3‧5 Project for Disciplines of Excellence at West China Hospital, Sichuan University (ZYJC20002), and the Sichuan Science and Technology Program (2024NSFSC1518).

## Author contributions

Chao Liu, Mengge Wang Huijun Yuan, and Guanglin He conceived and designed the study. Mengge Wang and Guanglin He collected the samples. Mengge Wang and Guanglin He extracted the genomic DNA and performed the genotyping. Yunhui Liu, Lintao Luo, Yuhang Feng, Zhiyong Wang, and Ting Yang performed the population genetic analysis. Mengge Wang, and Guanglin He drafted the article. Mengge Wang and Guanglin He revised the article.

## Declaration of interests

The authors declare no competing interests.

## STAR★Methods

### Key resources table

**Table:** 

REAGENT or RESOURCE	SOURCE	IDENTIFIER
**Deposited data**		

Y-STR and Y-SNP haplotype data	This study	YHRD: https://yhrd.org/ (YA004726 for TCD, YA004729 for TML, YA004613 for TQH, YA004223 for YLS, and YA004730 for SDJ). National Genomics Data Center: https://ngdc.cncb.ac.cn/bioproject/browse/PRJCA028381

**Software and algorithms**		

BWA v0.7.13	Li and Durbin^71^	http://bio-bwa.sourceforge.net; RRID: SCR_010910
Picard v3.0.0	N/A	http://broadinstitute.github.io/picard; RRID: SCR_006525
GATK v4.2.6.1.	McKenna et al.^72^	https://gatk.broadinstitute.org/hc/en-us; RRID: SCR_001876
BCFtools v1.8	Li^73^	https://www.htslib.org; RRID: SCR_005227
VCFtools	Danecek et al.^74^	https://vcftools.github.io/index.html; RRID: SCR_001235
GeneMapper ID v.1.5	N/A	https://www.thermofisher.com/order/catalog/product/4475073; RRID: SCR_014290
Chromas Lite V2.6.6	N/A	https://technelysium.com.au/wp/chromas/; RRID: SCR_000598
HaploGrouper	Jagadeesan et al.^75^	https://gitlab.com/bio_anth_decode/haploGrouper
the STR Analysis for Forensics (STRAF)	Gouy et al.^76^	https://straf-p7bdrhm3xq-ew.a.run.app/https://github.com/agouy/straf
YHRD website	N/A	https://yhrd.org/pages/tools/amova
SPSS v.25.0	N/A	https://www.ibm.com/support/pages/downloading-ibm-spss-statistics-25; RRID: SCR_002865
R v4.3.3	R CoreTeam^77^	https://cran.r-project.org/bin/windows/base/; RRID: SCR_001905
Arlequin v.3.5	Excoffier et al.^78^	https://cmpg.unibe.ch/software/arlequin35/; RRID: SCR_009051
MEGA v.7.0	Kumar et al.^79^	https://www.megasoftware.net/; RRID: SCR_000667
Surfer v.19.	Relethford^80^	https://www.goldensoftware.com/products/surfer/
MVSP v.3.22.	N/A	https://www.kovcomp.co.uk/downl2.html
Network 10.1	N/A	https://www.fluxus-engineering.com/sharenet.htm
Network Publisher	N/A	https://www.fluxus-engineering.com/sharenet.htm
Y-LineageTracker	Chen et al.^81^	https://github.com/Shuhua-Group/Y-LineageTracker
ArcMap	N/A	https://www.esri.com/en-us/arcgis/products/arcgis-desktop/overview
RaXML v8.0.0	Stamatakis et al.^82^	https://github.com/stamatak/standard-RAxML; RRID: SCR_006086
pathPhynder	Martiniano et al.^83^	https://github.com/ruidlpm/pathPhynder
BEAST v.1.10.4	Suchard et al.^84^	https://beast.community; RRID: SCR_010228
LogCombiner v1.10.4.	Drummond and Rambaut^85^	https://beast.community/logcombiner
Tracer v1.7	Rambaut et al.^86^	https://beast.community/tracer; RRID: SCR_019121
TreeAnnotator v1.10.4	Drummond and Rambaut^85^	https://beast.community/treeannotator
FigTree v1.4.4	N/A	http://tree.bio.ed.ac.uk/software/figtree/; RRID: SCR_008515

### Experimental model and study participant details

This study followed ethical standards set by the Medical Ethics Committees of West China Hospital of Sichuan University (Approval No. 2023-306) and the principles of the International Declaration of Helsinki. We collected samples in three batches. First, we obtained peripheral venous blood from 519 unrelated TB-speaking individuals in various communities after providing informed consent for genotyping STR and SNP profiles. This included 254 Tibetan individuals from multiple locations: 101 from Muli County, Liangshan Yi Autonomous Prefecture; 95 from Chengdu, Sichuan Province; and 58 from Qinghai Province. Additionally, we sampled 104 Yi participants from Liangshan Yi Autonomous Prefecture and 161 Sherpa participants from Dingjie County, Shigatse, Tibet Autonomous Region. We integrated these data with 3,779 previously reported genotypes from ethnolinguistically diverse Chinese populations to characterize general paternal profiles across China (Figures 1A and 1B). Second, we collected 72 representative samples from the D-Z31591 and O-CTS4658 lineages for whole-genome sequencing and merged them with 918 modern samples from the pilot work of the YangHuang cohort and 303 ancient Y chromosome sequences from published ancient autosome-based studies to elucidate the demographic dynamics of TB people further (Tables S15 and S16). Finally, we collected additional samples to explore the evolutionary history of the founding TB lineages. This included 918 samples from 31 provinces covering 275 prefecture-level cities associated with the D-Z31591 lineage and 3,388 samples from 34 provinces covering 373 prefecture-level cities linked to the O-CTS4658 lineage for high-density Y-SNP genotyping. The resource encompassed 37 ethnic groups and over four thousand ST-speaking individuals, including 3,811 Han Chinese individuals, 89 Tibetan individuals, 84 Yis individuals, 39 Huis individuals, 38 Manchus individuals, 27 Bais individuals, and 168 individuals from 31 other minority groups.

All participants provided informed consent, and the study procedures were approved by the Medical Ethics Committee of West China Hospital, Sichuan University (Approval No. 2023-1288). The study was conducted following the Human Genetic Resources Administration of China (HGRAC) guidelines and adhered to the principles of the 2013 revision of the Helsinki Declaration.

### Method details

#### Whole-genome sequencing and ancient Y chromosome sequences

The whole genomes of representative samples were sequenced using the DNBSEQ-T7 platform (MGI, Shenzhen, China) following an in-house protocol.^3^ We used BWA v0.7.13 ^71^ to map the raw sequencing reads to the GRCh37 human reference genome and Picard v3.0.0 to remove duplicate reads. Base quality score recalibration was performed using GATK v4.2.6.1. Y chromosome BAM files were extracted and combined with reference targeting sequencing 20 Mb Y chromosome BAMs.^72^ The GATK HaplotypeCaller, CombineGVCFs, and GenotypeGVCFs modules were used for the joint calling of genome-wide variants.^72^ We focused on high-quality Y chromosome regions, specifically the 10 Mb region used in Poznik’s population evolution modeling.^87^ Quality control was performed using BCFtools v1.8, filtering variants with missing call rates greater than 5%, base quality less than 20, and heterogeneity rates greater than 15%.^73^ Variants with missing call rates exceeding 5% were removed using VCFtools.^74^ The raw sequencing reads of ancient Tibetans were downloaded from the Genome Sequence Archive of the National Genomics Data Center (https://ngdc.cncb.ac.cn/gsa-human/) and aligned following standard ancient DNA research protocols.^41^ Quality-controlled BAM files were used for integrative analysis between modern and ancient genomic data and haplogroup classification.

#### Y-STR haplotype profiling

As a quality control measure, we used male DNA standard 9948 (Promega Corporation, USA) as a positive control throughout the study. For Y-STR haplotype profiling, we employed the AGCU Y37 Kit for multiplex amplification of 37 Y-STR loci.^88^ Ultrapure water served as the negative control. Each reaction mixture included 2 μL of reaction mixture, 1 μL of Y37 primers, 0.2 μL of DNA polymerase, and 1 μL of DNA template at 2 ng/μL, with the final volume adjusted to 5 μL using 0.8 μL of deionized water (ddH_2_O). Thermal cycling was conducted on a ProFlex 96-well PCR system (Thermo Fisher Scientific) under the following conditions: initial denaturation at 95°C for 2 min, 30 cycles of denaturation at 94°C for 30 s, annealing at 60°C for 1 min, extension at 72°C for 1 min, a final extension at 60°C for 20 min, and holding at 4°C. We analyzed the amplified products using an ABI 3500XL Genetic Analyzer. The electrophoresis setup included 9.8 μL of deionized formamide, 0.2 μL of AGCU Marker SIZ-500 internal standard, and 1 μL of either the amplified product or the Y37 allelic ladder standard. The electrophoresis parameters were an injection time of 10 s at 1.2 kV, followed by a 3-min prerun and a 22-min electrophoresis at 15 kV. Data interpretation was performed using GeneMapper ID v.1.5 software.

#### Sanger sequencing and detection of microvariant alleles

To identify microvariant alleles not cataloged in the standard Bin file, we used Sanger sequencing for validation. We used the DYS448 amplification primers from Hohoff et al.*,*^89^ the DYS570 and DYS627 primers from Ballantyne et al.*,*^35^ and the DYS527 primers from the NIST website (Table S1). The PCR amplification mixture included 10 μL of QIAGEN Multiplex PCR Master Mix (2×), 1 μL each of forward and reverse primers (10 μM), 2 μL of DNA template (2 ng/μL), and 6 μL of ddH_2_O. The PCR conditions, which varied by primer-specific annealing temperature, are detailed in Table S2. After amplification, we verified the specificity of the PCR products via polyacrylamide gel electrophoresis. We then sequenced the PCR products using the Sanger method to genotype the alleles accurately. Sequencing analyses were performed using Chromas Lite V2.6.6 software (Technelysium Pty Ltd., Australia), ensuring precise allele identification.

#### SnaPshot-based Y-SNP genotyping, microarray genotyping, and haplogroup classification

We genotyped 215 Y-SNP loci using SNaPshot panels (Figure 2A) following protocols described by Wang et al*.*^90^ Y-SNP profiles were analyzed with GeneMapper ID v.1.5 software. High-density Y-SNPs from 918 D-Z31591 and 3,388 O-CTS4658 samples were genotyped using the Thermo Fisher Scientific Illumina 23MF_v1 array, which includes 769,530 SNPs, 27,280 of which are phylogenetically informative Y chromosome SNPs. We manually classified haplogroups for 215 Y-SNP-based genotypes and used Haplogrouper^75^ for haplogroup inference on high-density Y-SNP data and whole Y chromosome sequences, adhering to the Y-DNA Haplogroup Tree 2019–2020 standards.

### Data analysis

#### Y-STR data analysis

For our Y-STR data analysis, the allele frequencies and genetic diversity of each Y-STR locus were calculated using the STR Analysis for Forensics (STRAF) software.^76^ To ensure data clarity, three multicopy loci—DYS527, DYS385a/b, and DYF387S1—were excluded from the analysis. Furthermore, the allele count for DYS389II was adjusted by subtracting DYS389I to derive DYS389b. Allele frequency was computed using the direct counting method for multicopy loci, copy number variations, and null alleles. The frequency of each Y-STR haplotype was calculated using the formula =x/N, where *x* represents the number of observed haplotypes and *N* is the total sample size. HD, GD, HMP, and DC were derived using the following formulas: *HD*/*GD* = $N\times \left(1-\sum_{i=1}^{k}\;pi^{2}\right)/\left(N-1\right)$, *HMP* $=\sum_{i=1}^{k}\;pi^{2}$, and *DC* = $k/\sum_{i=1}^{k}\;\left(pi\times N\right)$. Here, *pi* is the frequency of the i-th haplotype, *k* is the number of haplotypes, and *N* is the sample size of each studied population.

To evaluate genetic distances among geographically diverse populations, we analyzed 29 Y-STRs cataloged in the Y Chromosome Haplotype Reference Database (YHRD), including subsets of 27 Y-STRs from the Yfiler Plus kit and 17 Y-STRs from the Yfiler kit. We estimated genetic distances (Rst) using the AMOVA&MDS tool on the YHRD website. The resulting Rst genetic distance matrix was subjected to multidimensional scaling (MDS) analysis in SPSS v.25.0 and visualized using R software^77^ to enhance the interpretability of genetic relationships. Detailed descriptions of the reference populations used in this analysis are provided in Tables S3 and S4.^42^^,^^43^^,^^44^^,^^91^ We performed an analysis of molecular variance (AMOVA) with Arlequin v.3.5 to assess molecular variance within and between these populations.^78^ Additionally, we constructed a neighbor-joining (NJ) phylogenetic tree based on the Rst matrix using MEGA v.7.0 ^79^ to further elucidate phylogenetic relationships.

#### Y-SNP data analysis

We calculated haplogroup frequencies within the studied populations using the direct counting method. HGD was determined by the formula *HGD* = $\times \left(1-\sum_{i=1}^{k}\;pi^{2}\right)/\left(N-1\right)$, where *pi* represents the frequency of the i-th haplogroup, *k* is the total number of observed haplogroups, and *N* is the sample size. To visualize the distribution of major haplogroups among the TB populations, we generated contour maps using Surfer v.19.^80^ Genetic distances (Fst) between geographically distinct populations were calculated based on either 113 Y-SNPs common among worldwide populations or 157 Y-SNPs common among Chinese populations using Arlequin v.3.5. We conducted MDS analysis of the Fst genetic distance matrix with SPSS v.25.0 to determine spatial genetic relationships. Additionally, we performed principal component analysis (PCA) based on haplogroup frequencies using MVSP v.3.22. Phylogenetic relationships were further delineated through an NJ phylogenetic tree constructed with MEGA v.7.0. AMOVA based on Y-SNP haplotypes was conducted using Arlequin v.3.5 to determine variance components attributed to different levels of population grouping.

#### Integrative analysis of Y-SNPs and Y-STRs

To elucidate genetic relationships among populations, we constructed a median-joining (MJ) network using Network 10.1 and Network Publisher software, integrating Y-SNP-STR haplotypes. To enhance analysis accuracy, we excluded DYS385a/b due to its multicopy nature and treated DYS389 as two separate loci: DYS389I and DYS389b (calculated as DYS389II−DYS389I). For DYF387S1, we considered only the DYF387S1b allele. In this network analysis, we assigned Y-SNPs a high weight of 99 due to their lower mutation rates, providing stability to the network structure. Conversely, Y-STRs, with greater variability, were assigned weights ranging from 1 to 5, inversely proportional to their mutation rates. This weighting system balanced the contributions of SNPs and STRs, offering a detailed and nuanced view of the genetic landscape and historical population dynamics.^92^

#### Spatial correlation analysis

We conducted spatial correlation analysis using R software and Y-LineageTracker,^81^ applying parameters such as –level and –freq to estimate haplogroup frequencies at both the provincial and prefectural levels. We examined the geographical distribution and potential phylogeographic origins of founding lineages through spatial autocorrelation analysis performed in ArcMap.

#### Whole Y chromosome sequence-based demographical history reconstruction

We obtained high-quality variant calls using the sequence masks and filters mentioned above, which served as high-confidence 10 Mb targeted Y chromosome regions. The final dataset of 994 samples was used to construct a maximum-likelihood tree via RaXML v8.0.0,^82^ with 200 rapid bootstrap inferences and a maximum-likelihood search. We then integrated 303 ancient Y chromosome sequences into the reconstructed reference phylogeny for combined analysis using pathPhynder.^83^ Coalescent times for each node were estimated using Bayesian Markov Chain Monte Carlo (MCMC) methods with BEAST v.1.10.4 software.^84^ To preserve the phylogenetic topology, we included additional samples from other haplogroups, specifically four samples from haplogroup B, to root the tree.^93^ We conducted four parallel runs, each with a different seed number, and merged them using LogCombiner v1.10.4.^85^ Each run consisted of 60 million chains, logging every 3,000 steps. The results were manually inspected using Tracer v1.7 software^86^ and the initial 25% was discarded as burn-in using TreeAnnotator v1.10.^85^ Consistent parameters were maintained across all runs, including the GTR substitution model with Gamma and Invariant sites heterogeneity model, a strict clock with a uniform distribution prior to mutation rate (7.4e-10; 95% CI: 6.7e-10-8.6e-10 mutations/nucleotide/year), and the Bayesian Skyline model with a group size of 10. The NO-M214 node served as the calibration point for estimating coalescence age, with an age of 41,900 years (95% CI: 40,175–43,591).^94^ The maximum clade credibility tree was then visualized using FigTree.

### Quantification and statistical analysis

We conducted a Pearson correlation analysis using R software between the founding lineage frequency and the geographical coordinates and ADMIXTURE-based admixture proportion in the Figure 5C, where ∗ represents 0.05 < *p* value <0.01, ∗∗ represents 0.01 ≤ *p* value <0.001, ∗∗∗ represents *p* value <0.001. Meanwhile, we conducted another Pearson correlation analysis between the frequency of two founding lineages and the latitude and longitude coordinates in the Figure 5D, where the R value is considered significant if the *p*-value is less than 0.05.

## Supplemental information

Document S1. Figures S1–S17 and Tables S1, S2, S5, S8, and S14

Table S3. Reference populations for population comparative analysis based on 29 Y-STR Loci, related to STAR Methods

Table S4. Reference population information for Y-SNP-STR combined analysis, related to STAR Methods

Table S6. Allele Frequency Distribution of Single-Copy Y-STRs in Five Studied Populations, related to STAR Methods

Table S7. Genotype Frequency Distribution of Multi-Copy Y-STRs in Five Studied Populations, related to STAR Methods

Table S9. Global genetic distances estimated from 17 Y-STR loci typing data, related to Figure S3

Table S10. Genetic distances among Chinese populations estimated from 29 Y-STR loci typing data, related to Figure S4 and S6

Table S11. Genetic distances among Chinese populations estimated from 27 Y-STR loci typing data, related to Figure S5

Table S12. Genetic distances among global populations estimated from 113 Y-SNP loci typing data, related to Figure S8

Table S13. Genetic distances among Chinese populations estimated from 157 Y-SNP loci typing data, related to Figure S9

Table S15. The detailed information of modern populations with whole Y chromosome sequences in this study, related to Figure 1, Figure 2 and STAR Methods

Table S16. The detailed information of ancient populations in this study, related to Figure 1, Figure 2 and STAR Methods
