# Supplementary material for: Genomic insights into Neolithic founding paternal lineages around the Qinghai-Xizang Plateau using integrated YanHuang resource
Source: iScience. 2024 Nov 22;27(12):111456. doi: 10.1016/j.isci.2024.111456 (PMC11696643; doi:10.1016/j.isci.2024.111456)
Supplement: Document S1. Figures S1–S17 and Tables S1, S2, S5, S8, and S14 [file mmc1.pdf]

iScience, Volume *27*

Supplemental information

Genomic insights into Neolithic founding paternal lineages around the Qinghai-Xizang Plateau using integrated YanHuang resource

Mengge Wang, Yunhui Liu, Lintao Luo, Yuhang Feng, Zhiyong Wang, Ting Yang, Huijun Yuan, Chao Liu, and Guanglin He

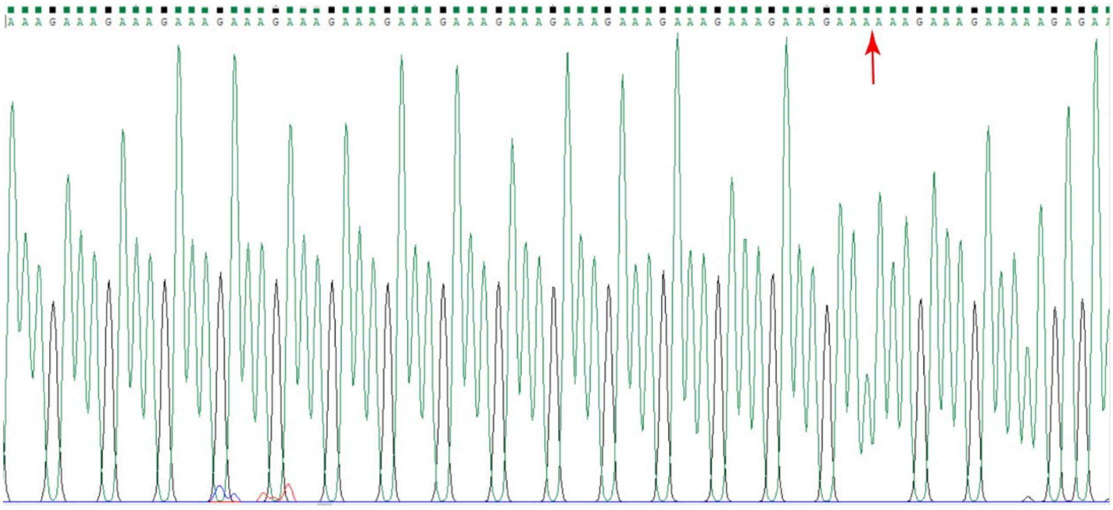

Fig. S1. The Sanger sequencing results of the DYS627 locus and its flanking sequences.

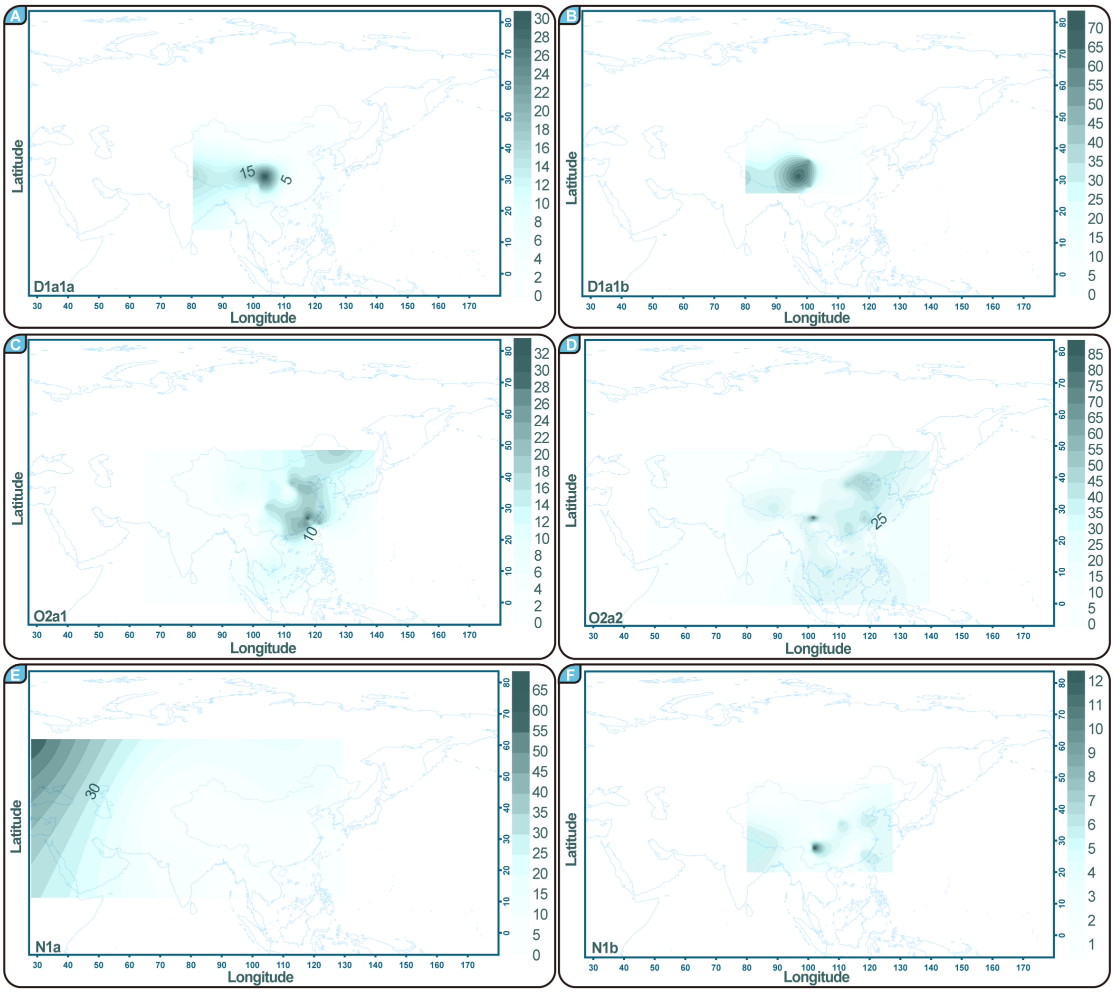

Fig. S2. Spatial frequency distribution of major haplogroups among newly genotyped TB-speaking populations. Contour map of haplogroup frequencies of D1a1a (A), D1a1b (B), O2a1 (C), O2a2 (D), N1a (E), and N1b (F). This figure illustrates the geographical spread of major haplogroups across the regions occupied by TB-speaking populations and relevant reference groups.

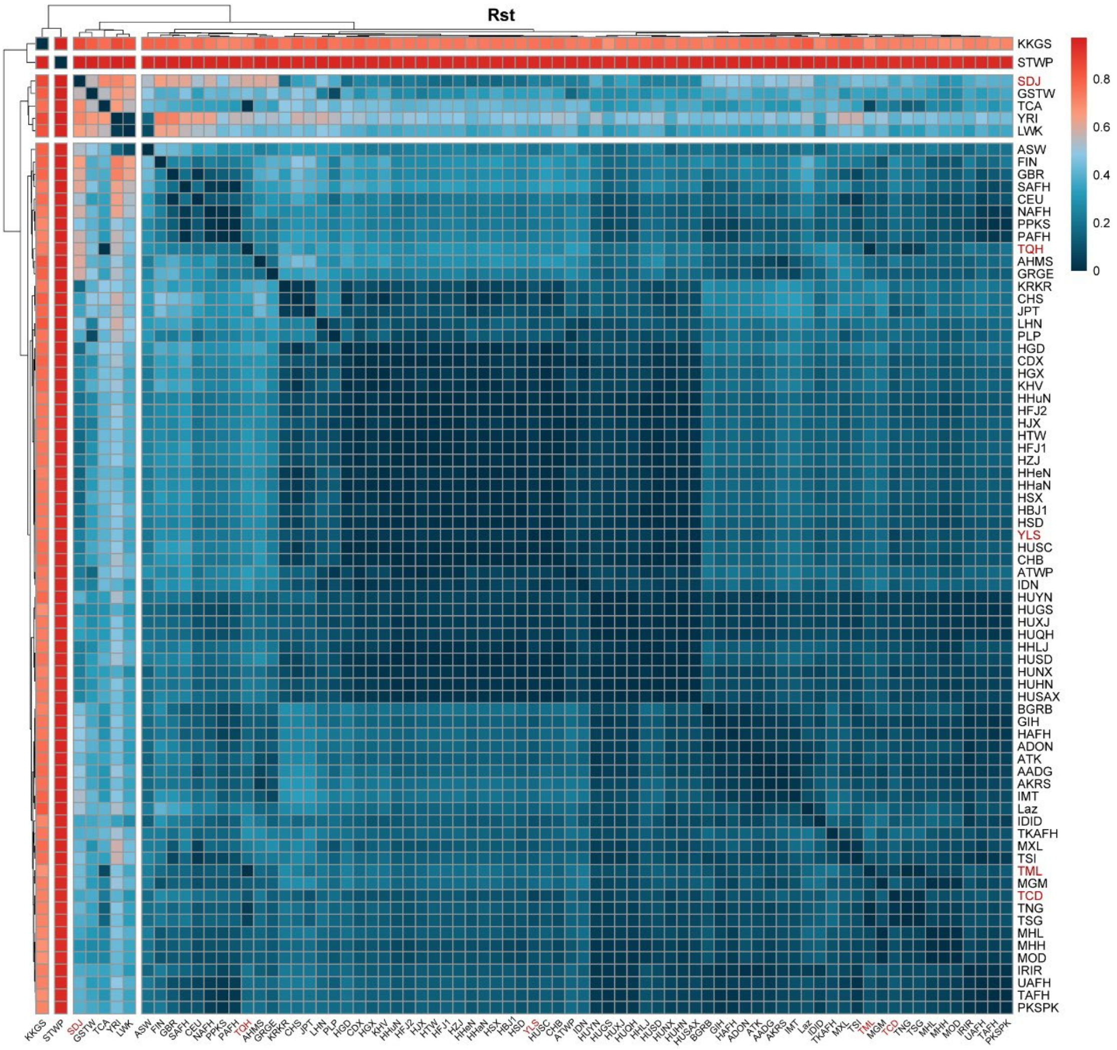

Fig. S3. The genetic distances between worldwide populations estimated based on the haplotypes of 17 Y-STRs. The newly collected Tibeto-Burman (TB)-speaking populations are indicated in red font. KKGS: Kyrgyz_Kyrgyzstan; STWP: Sinitic_PlainTribes; SDJ: Sherpa_Dingjie; GSTW: Gaoshan_Taiwan; TCA: Tibetan_Chamdo; YRI: Yoruba_Ibadan; LWK: Luhya_Kenya; ASW: African_Americans_SWUSA; FIN: Finnish; GBR: British; SAFH: Pathan_Afghanistan_South; CEU: European_Utah; NAFH: Pathan_Afghanistan_North; PPKS: Pathan_Pakistan; PAFH: Pathan_Afghanistan; TQH: Tibetan_Qinghai; AHMS: Armenian_Hemsheni; GRGE: Georgians_Eastern; KRKR: Korean; CHS: Han_Southern; JPT: Japanese_Tokyo; LHN: Li_Hainan; PLP: Philippinese; HGD: Han_Guangdong; CDX: Dai_Xishuangbanna; HGX: Han_Guangxi; KHV: Kinh_HoChiMinh; HHuN: Han_Hunan; HFJ2: Han_Fujian2; HJX: Han_Jiangxi; HTW: Han_Taiwan; HFJ1: Han_Fujian1; HZJ: Han_Zhejiang; HHeN: Han_Henan; HHaN: Han_Hainan; HSX: Han_Shanxi; HBJ1: Han_Beijing1; HSD: Han_Shandong; YLS: Yi_Liangshan; HUSC: Hui_Chengdu; CHB: Han_Beijing2; ATWP: Austronesian_PlainTribes; IDN: Indonesian; HUYN: Hui_Yunnan; HUGS: Hui_Lanzhou; HUXJ: Hui_Xinjiang; HUQH: Hui_Qinghai; HHLJ: Han_Heilongjiang; HUSD: Hui_Shandong; HUNX: Hui_Wuzhong; HUHN: Hui_Henan; HUSAX: Hui_Shaanxi; BGRB: Bulgarian_Bulgaria; GIH: Gujarati_Indian_TX; HAFH: Hazara_Afghanistan; ADON: Armenian_Don; ATK: Armenian_Erzurum; AADG: Armenian_Adygei; AKRS: Armenian_Krasnodar; IMT: Imeretins; Laz: Laz; IDID: Indian_India; TKAFH: Turkmen_Afghanistan; MXL: Mexican_American_LosAngeles; TSI: Toscani_Italy; TML: Tibetan_Muli; MGM: Mongolian_Mongolia; TCD: Tibetan_Chengdu; TNG: Tibetan_Ngari; TSG: Tibetan_Shigatse; MHL: Mongolian_Hulunbuir; MHH: Mongolian_Hohhot; MOD: Mongolian_Ordos; IRIR: Iranian_Iran; UAFH: Uzbek_Afghanistan; TAFH: Tajik_Afghanistan; PKSPK: Pakistani_Pakistan.

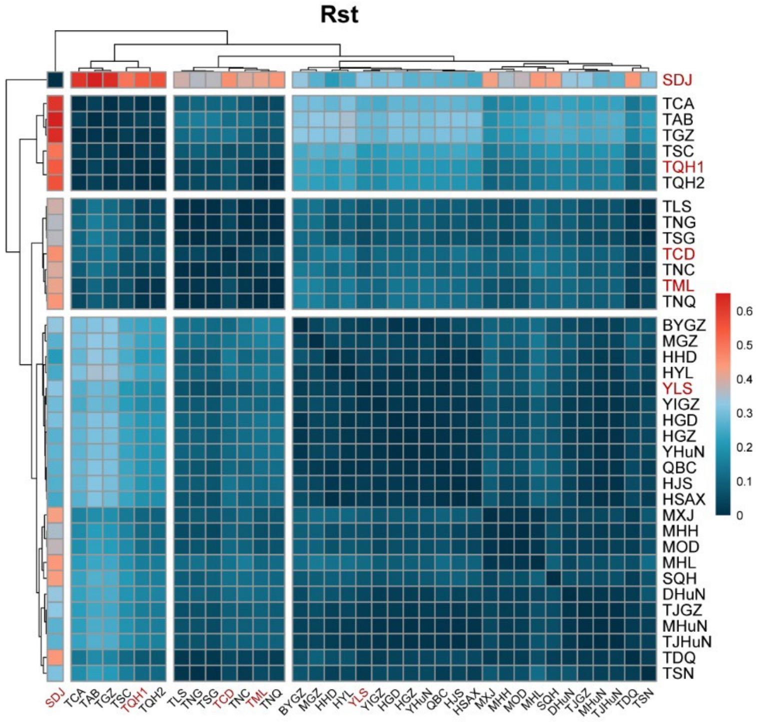

Fig. S4. Genetic distances among Chinese populations estimated based on the haplotypes of 29 Y-STR loci. The newly collected TB-speaking populations are indicated in red font. SDJ: Sherpa_Dingjie; TCA: Tibetan_Chamdo; TAB: Tibetan_Ngawa; TGZ: Tibetan_Garzê; TSC: Tibetan_Sichuan; TQH1: Tibetan_Qinghai1; TQH2: Tibetan_Qinghai2; TLS: Tibetan_Lhasa; TNG: Tibetan_Ngari; TSG: Tibetan_Shigatse; TCD: Tibetan_Chengdu; TNC: Tibetan_Nyingchi; TML: Tibetan_Muli; TNQ: Tibetan_Nagqu; BYGZ: Buyei_Guizhou; MGZ: Miao_Guizhou; HHD: Han_Handan; HYL: Han_Yulin; YLS: Yi_Liangshan; YIGZ: Yi_Guizhou; HGD: Han_Guangdong; HGZ: Han_Guizhou; YHuN: Yao_Hunan; QBC: Qiang_Beichuan; HJS: Han_Jiangsu; HSAX: Han_Shaanxi; MXJ: Mongolian_Xinjiang; MHH: Mongolian_Hohhot; MOD: Mongolian_Ordos; MHL: Mongolian_Hulunbuir; SQH: Salar_Qinghai; DHuN: Dong_Hunan; TJGZ: Tujia_Guizhou; MHuN: Miao_Hunan; TJHuN: Tujia_Hunan; TDQ: Tibetan_Diqing; TSN: Tibetan_Shannan.

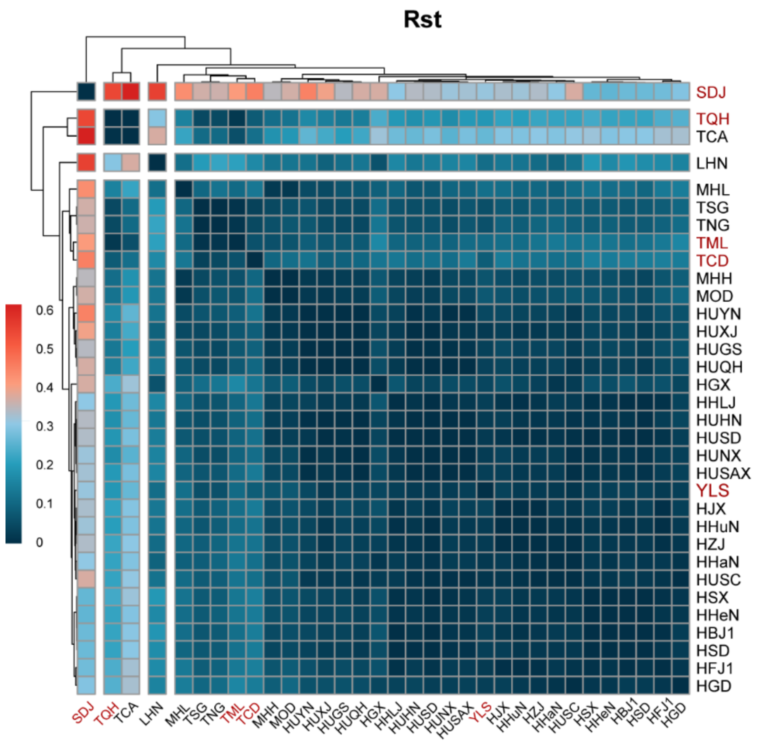

Fig. S5. Genetic distances among Chinese populations estimated based on the haplotypes of 27 Y-STRs. The newly collected TB-speaking populations are indicated in red font. SDJ: Sherpa_Dingjie; TQH: Tibetan_Qinghai; TCA: Tibetan_Chamdo; LHN: Li_Hainan; MHL: Mongolian_Hulunbuir; TSG: Tibetan_Shigatse; TNG: Tibetan_Ngari; TML: Tibetan_Muli; TCD: Tibetan_Chengdu; MHH: Mongolian_Hohhot; MOD: Mongolian_Ordos; HUYN: Hui_Yunnan; HUXJ: Hui_Xinjiang; HUGS: Hui_Lanzhou; HUQH: Hui_Qinghai; HGX: Han_Guangxi; HHLJ: Han_Heilongjiang; HUHN: Hui_Henan; HUSD: Hui_Shandong; HUNX: Hui_Wuzhong; HUSAX: Hui_Shaanxi; YLS: Yi_Liangshan; HJX: Han_Jiangxi; HHuN: Han_Hunan; HZJ: Han_Zhejiang; HHaN: Han_Hainan; HUSC: Hui_Chengdu; HSX: Han_Shanxi; HHeN: Han_Henan; HBJ1: Han_Beijing1; HSD: Han_Shandong; HFJ1: Han_Fujian1; HGD: Han_Guangdong.

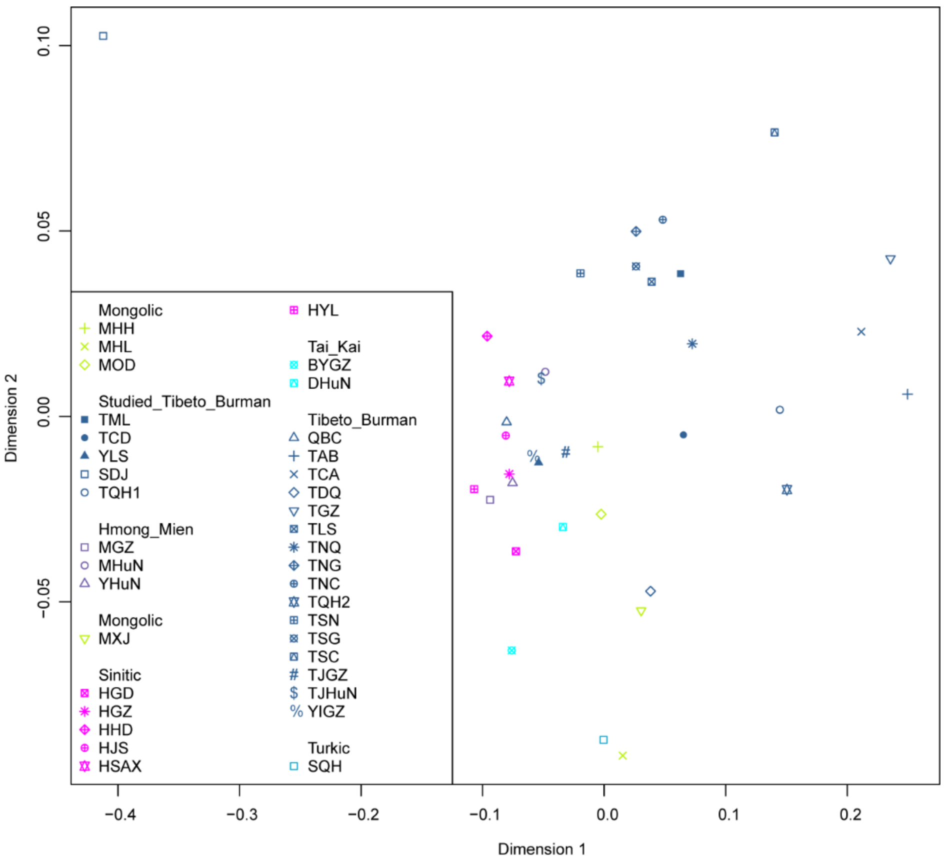

Fig. S6. The results of multidimensional scaling (MDS) analysis between the newly studied populations and 32 Chinese reference populations based on Rst genetic distances. The 29-Y-STR-based Rst genetic distances are used here. The population abbreviations are the same as in **Fig. S4**.

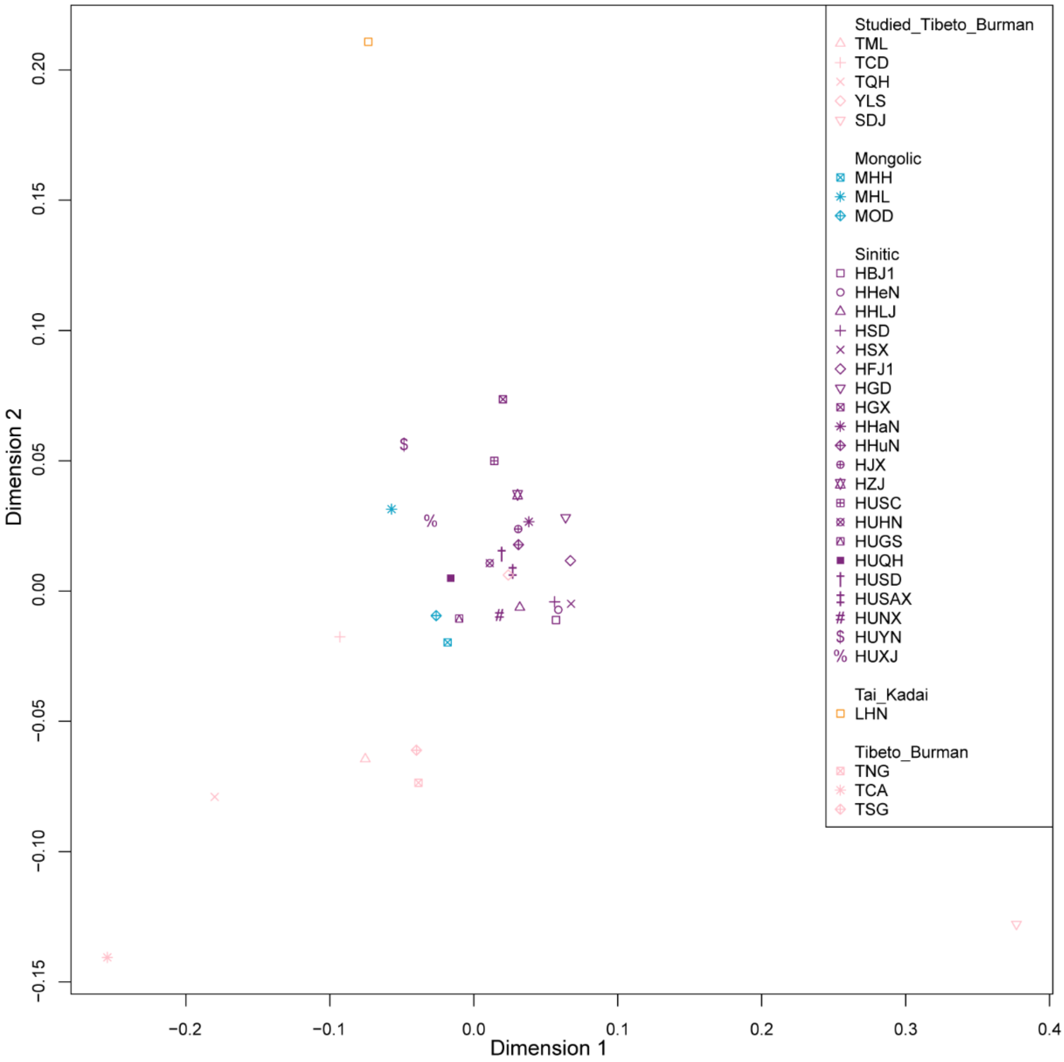

Fig. S7. The MDS results between the newly studied populations and 28 Chinese reference populations based on Rst genetic distances. The 27-Y-STR-based Rst genetic distances are used here. The population abbreviations are the same as in **Fig. S5**.

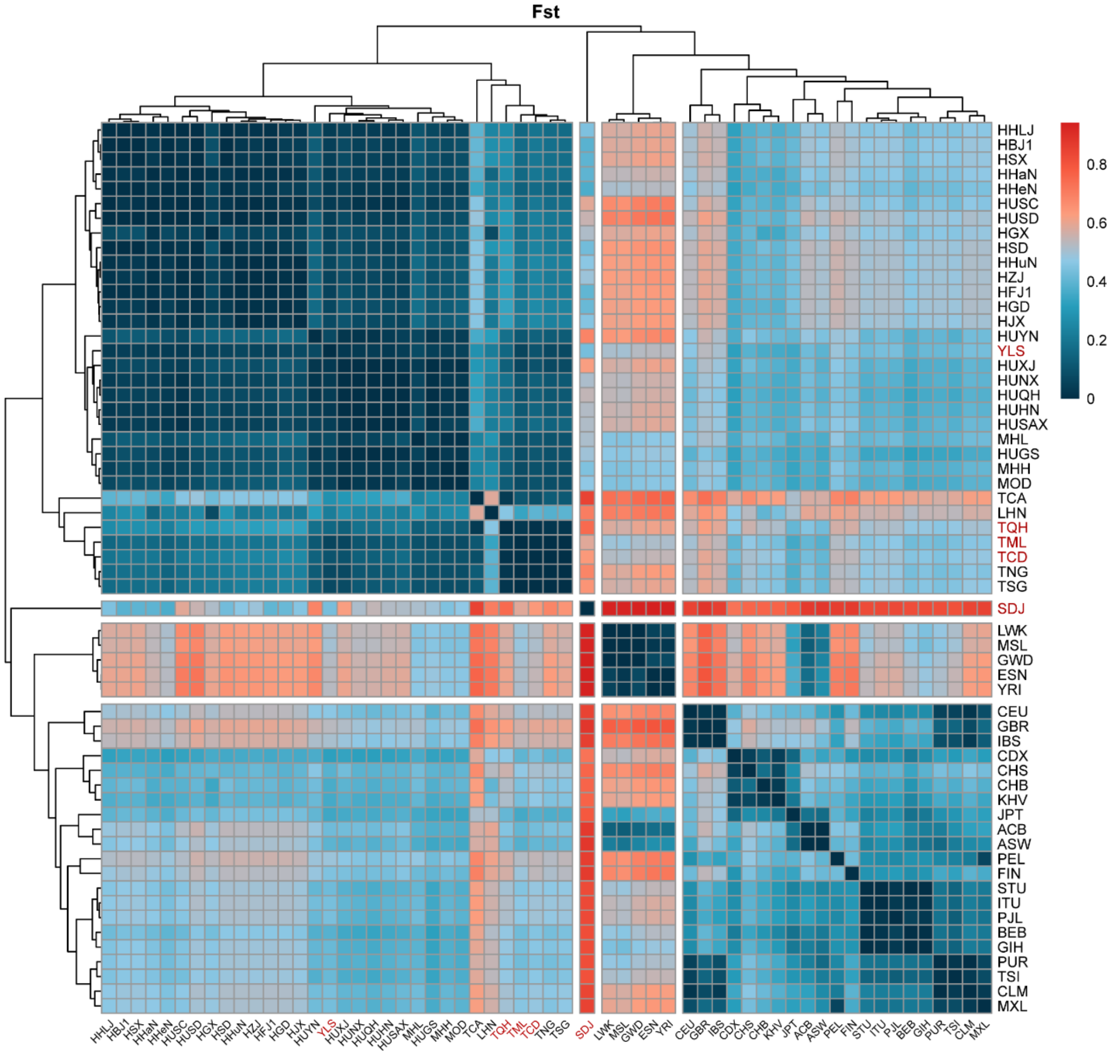

Fig. S8. Genetic distances among global populations estimated based on the haplotypes of 113 Y-SNPs. The newly collected TB-speaking populations are indicated in red font. The population abbreviations are the same as in **Fig. S3**.

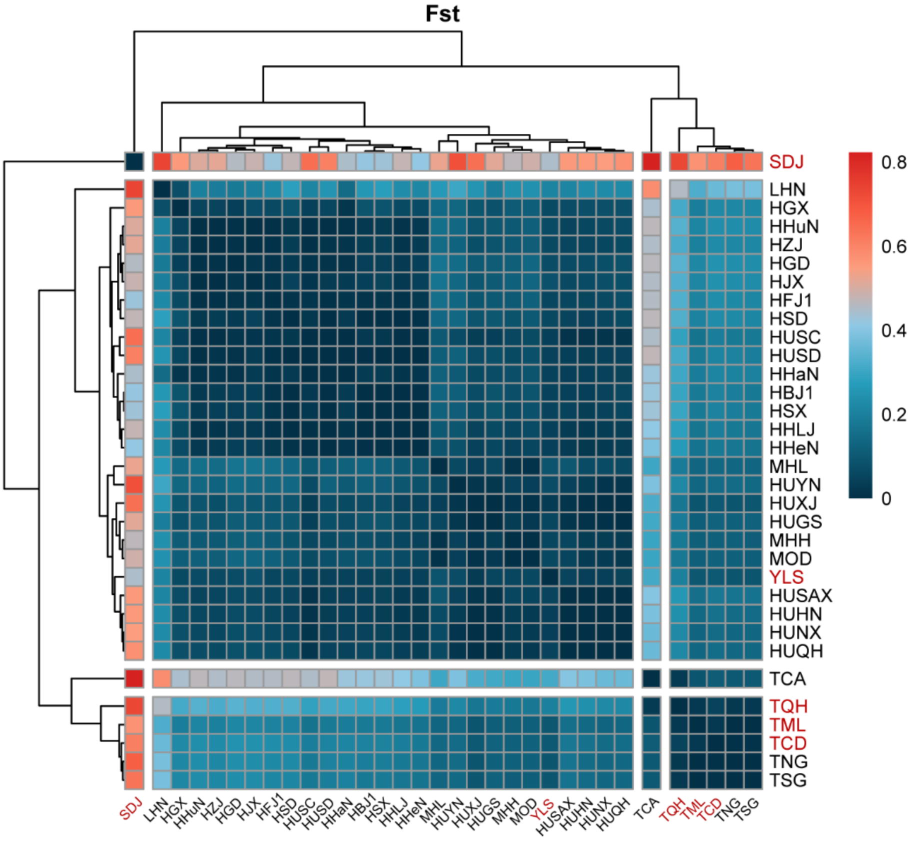

Fig. S9. Genetic distances among Chinese populations estimated based on the haplotypes of 157 Y-SNPs. The newly genotyped TB-speaking populations are indicated in red font. The population abbreviations are the same as in **Fig. S5**.

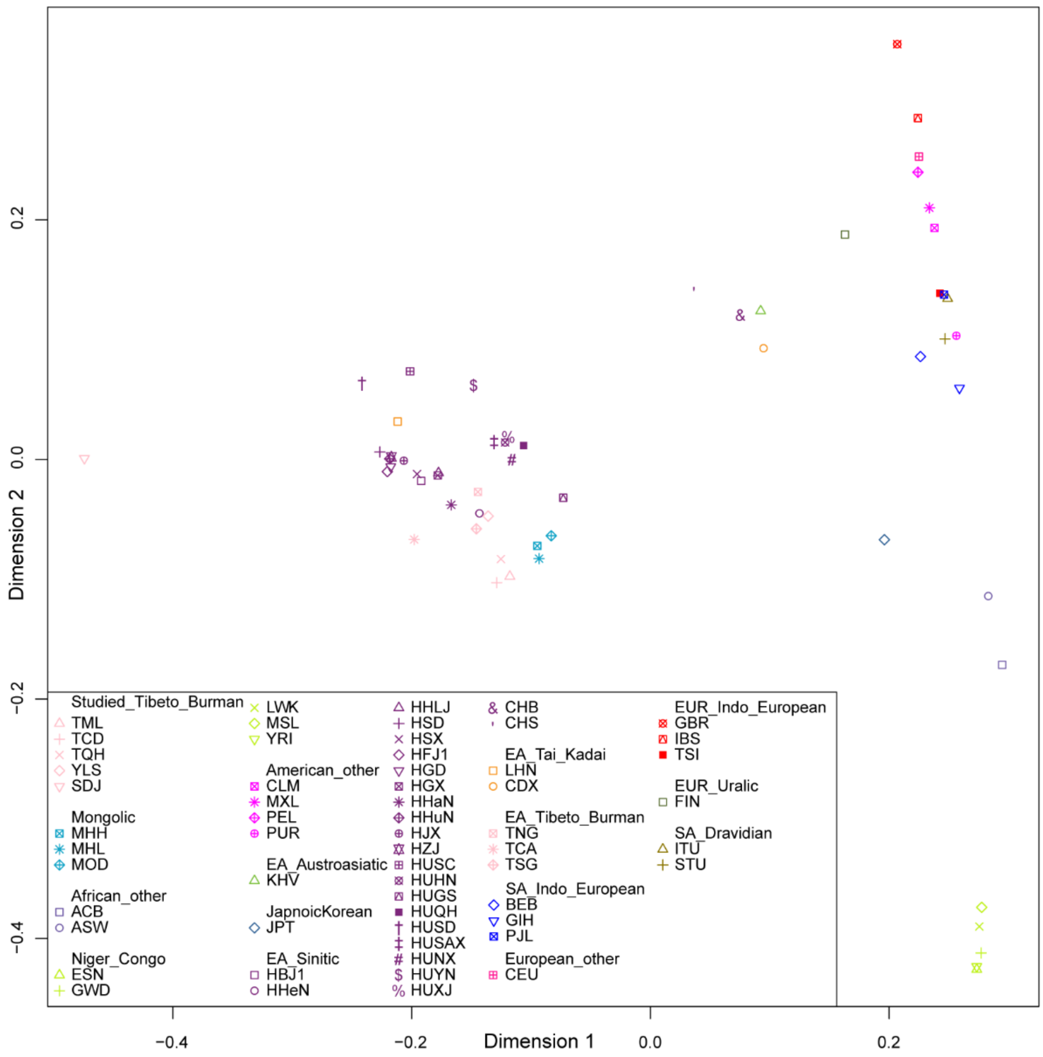

Fig. S10. The MDS results between the newly studied populations and 54 global reference populations based on Fst genetic distances. The 113-Y-SNP-based Fst genetic distances are used here. The population abbreviations are the same as in **Fig. S3**.

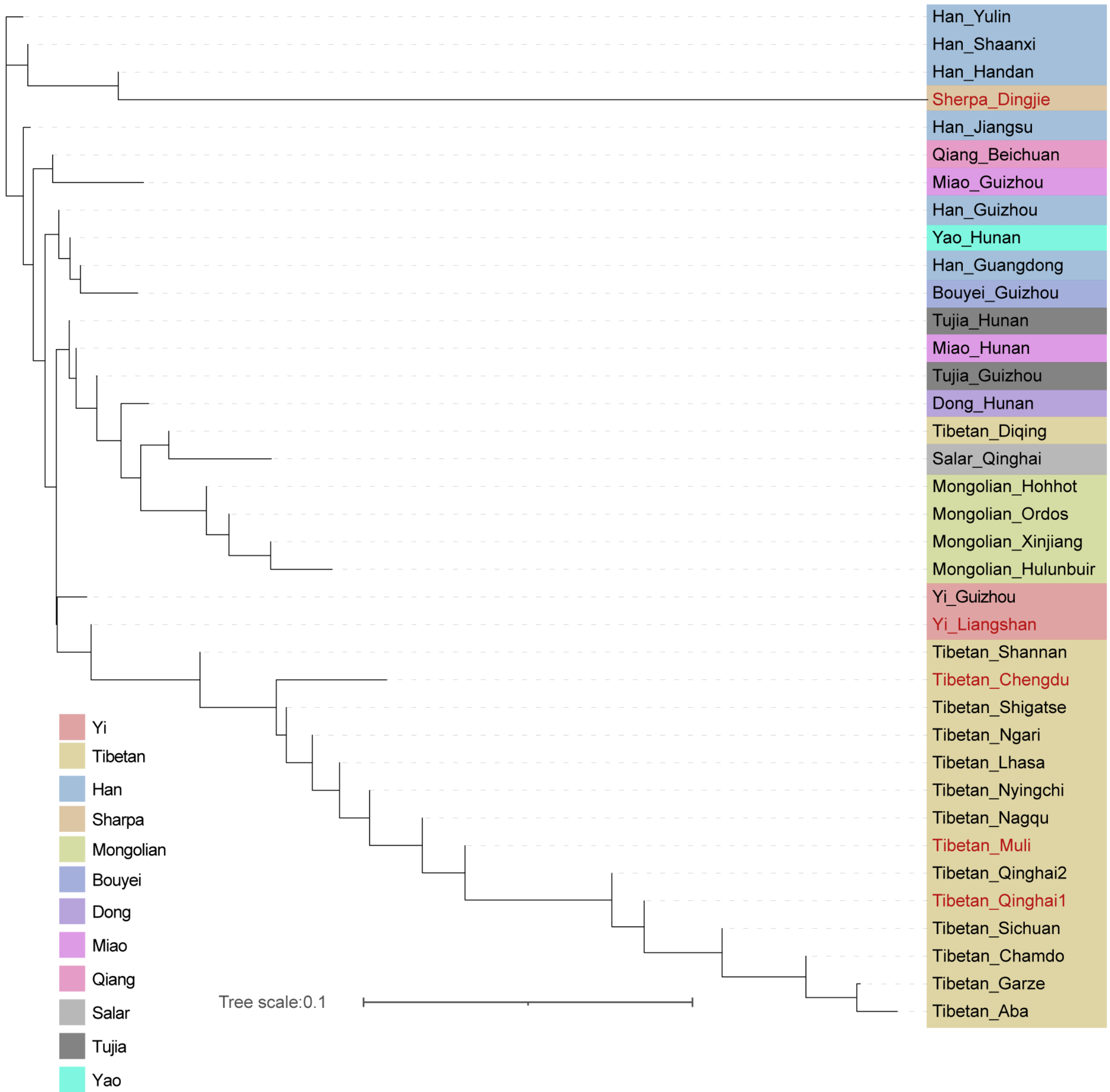

Fig. S11. The neighbor-joining (NJ) phylogenetic tree constructed from the Rst genetic distance matrix based on 29 Y-STR haplotypes. The newly collected TB-speaking populations are indicated in red font.

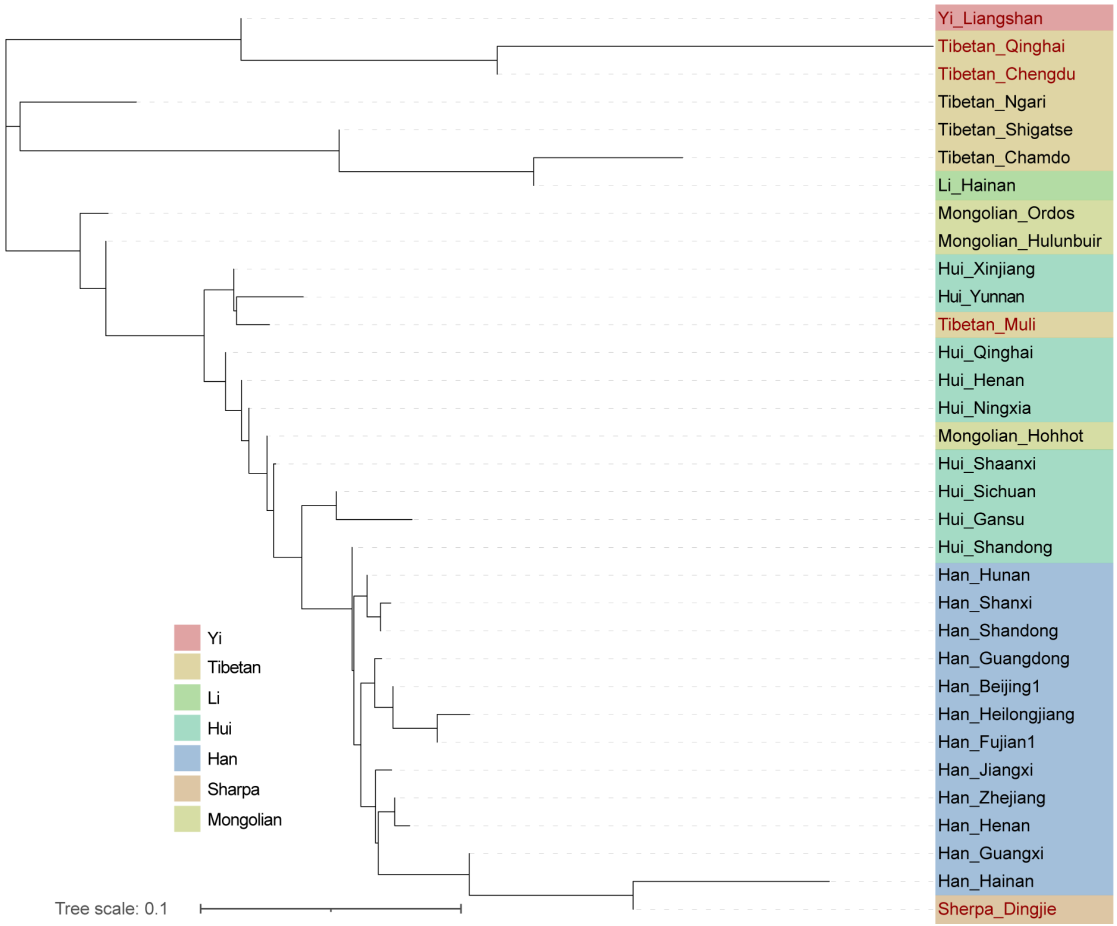

Fig. S12. The NJ phylogenetic tree constructed from the Fst genetic distance matrix based on Y-SNP haplotypes. The newly genotyped TB-speaking populations are indicated in red font.

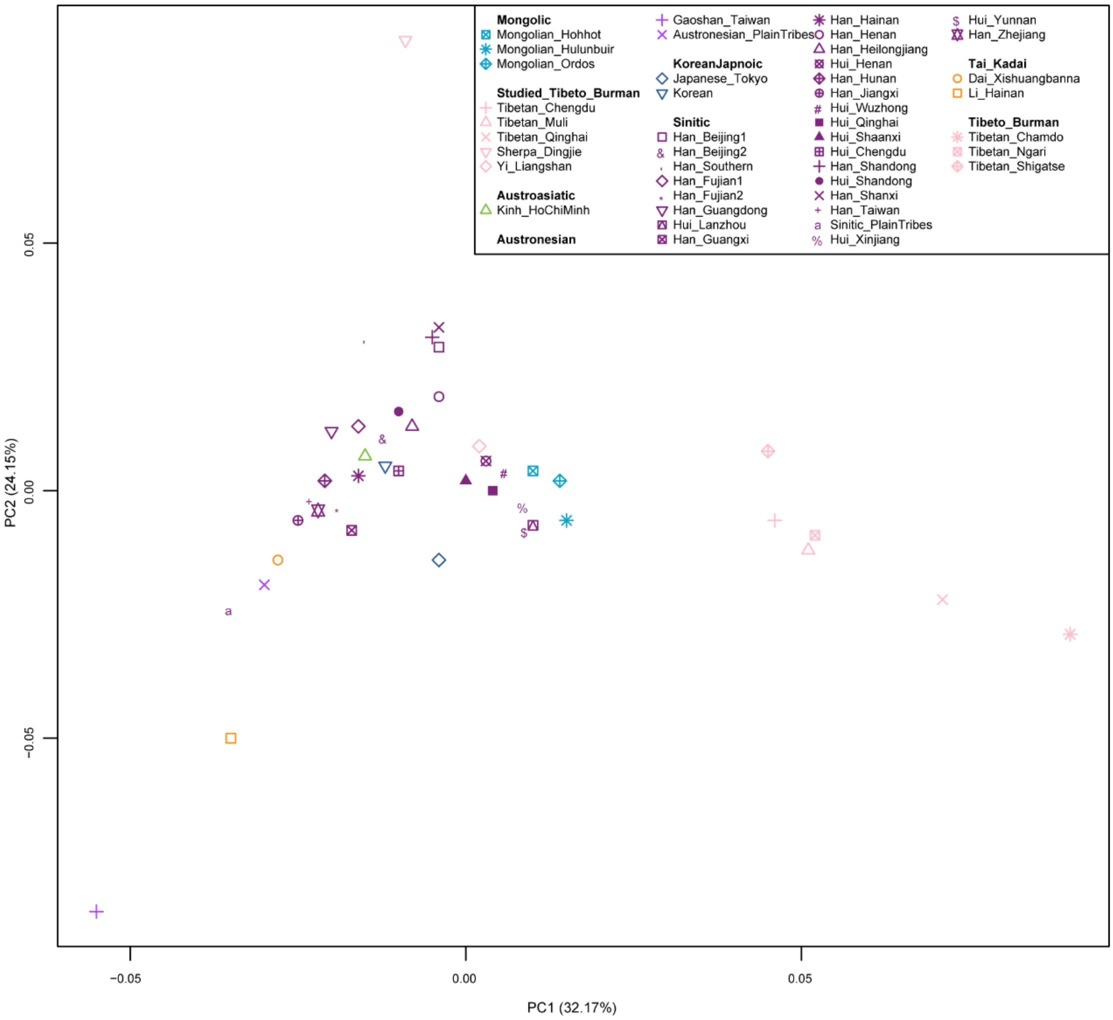

Fig. S13. The results of the principal component analysis between the newly studied populations and 39 East Asian reference populations.

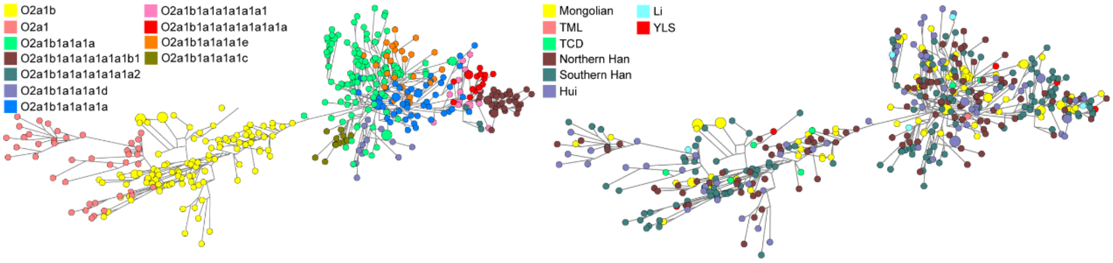

Fig. S14. The median-joining (MJ) network-based topology of O2a1-L467 and its subhaplogroups.

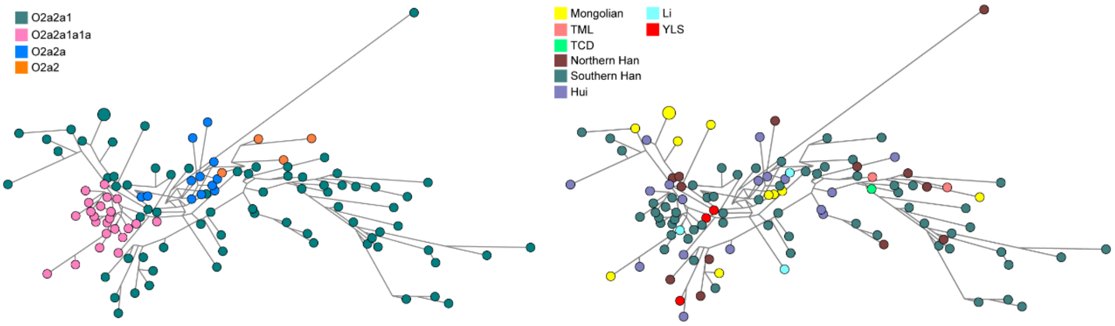

Fig. S15. The MJ network-based topology of O2a2a-M188 and its subhaplogroups.

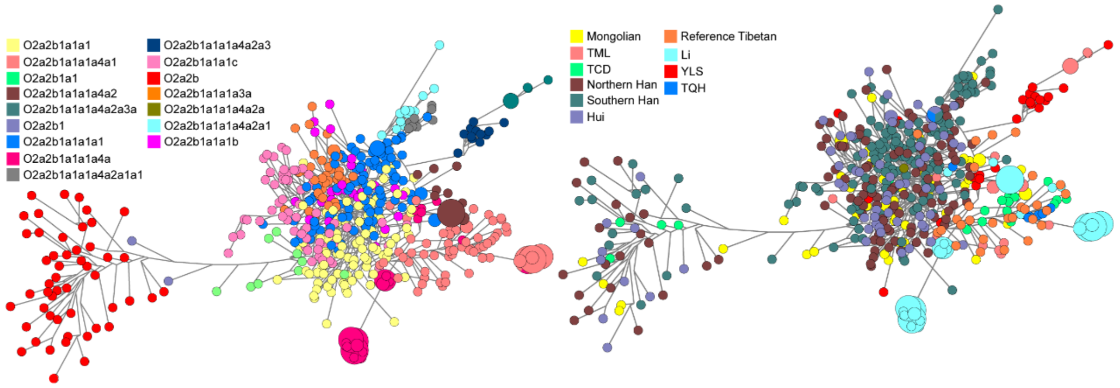

Fig. S16. The MJ network-based topology of O2a2b1a1-M117 and its subhaplogroups.

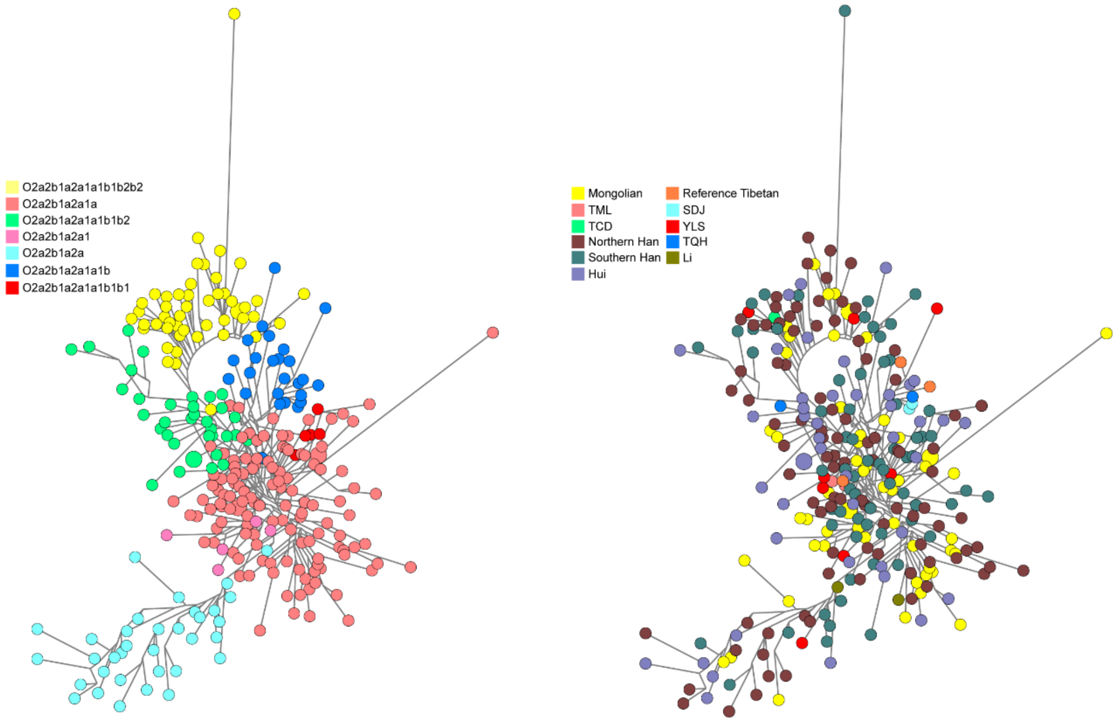

Fig. S17. The MJ network-based topology of O2a2b1a2a-F444 and its subhaplogroups.

Table S1. Amplification primer sequences for Y-STRs with microvariant alleles, related to STAR Methods

Y-STR		Primer
DYS448	F	TGTCAAAGAGCTTCAATGGAGA
	R	TCTTCCTTAACGTGAATTTCCTC
DYS570	F	GAACTGTCTACAATGGCTCACG
	R	TCAGCATAGTCAAGAAACCAGACA
DYS627	F	CTAGGTGACAGCGCAGGATT
	R	GGATAATGAGCAAATGGCAAG
DYS527	F	TCGCAAACATAGCACTTCAG
	R	TTCTAGGAAGATTAGCCACAACA

Table S2. PCR amplification conditions for Y-STRs with microvariant alleles, related to STAR Methods

PCR Amplification Conditions I for DYS627,		
**Reaction Stage**	**Temperature**	**Reaction Time**
Initial Denaturation	95℃	10min
	94℃	30 s
10 Cycles	60-50℃ (-1℃/Cycle)	30 s
	72℃	45 s
	94℃	30 s
25 Cycles	50℃	30 s
	72℃	45 s
Final Extension	60℃	45 min
Completion	15℃	Hold

PCR Amplification Conditions II for DYS448 and DYS527		
**Reaction Stage**	**Temperature**	**Reaction Time**
Initial Denaturation	95℃	10min
	94℃	30 s
10 Cycles	65-55℃ (-1℃/Cycle)	30 s
	72℃	45 s
	94℃	30 s
25 Cycles	55℃	30 s
	72℃	45 s
Final Extension	60℃	45 min
Completion	15℃	Hold

PCR Amplification Conditions III for DYS570		
**Reaction Stage**	**Temperature**	**Reaction Time**
Initial Denaturation	95℃	15min
	94℃	30 s
20 Cycles	70-50℃ (-1℃/Cycle)	45 s
	72℃	60 s
	94℃	30 s
15 Cycles	50℃	30 s
	72℃	45 s
Final Extension	60℃	45 min
Completion	15℃	Hold

Table S5. Forensic parameters of different Y-STR amplification systems, related to STAR Methods

Population	Haplotype Diversity				Haplotype Match Probability				Discrimination Power			
	MHT	Yfiler	Yfiler Plus	AGCU Y37	MHT	Yfiler	Yfiler Plus	AGCU Y37	MHT	Yfiler	Yfiler Plus	AGCU Y37
Tibetan_Muli	0.9784	0.9952	0.9978	0.9978	0.0313	0.0146	0.0121	0.0121	0.6733	0.8119	0.9109	0.9109
Tibetan_Chengdu	0.9657	0.9982	0.9998	0.9998	0.0444	0.0123	0.0107	0.0107	0.6947	0.9263	0.9895	0.9895
Tibetan_Qinghai	0.9758	0.9976	1.0000	1.0000	0.0410	0.0196	0.0172	0.0172	0.7241	0.9483	1.0000	1.0000
Yi_Liangshan	0.9959	0.9983	0.9998	1.0000	0.0137	0.0113	0.0098	0.0096	0.8558	0.9423	0.9904	1.0000
Sherpa_Dingjie	0.9344	0.9913	0.9981	0.9988	0.0714	0.0149	0.0081	0.0074	0.3478	0.6646	0.8944	0.9193

MHT: Minimal Haplotype Core Y-STR Set

Table S8. The distributions of null alleles, microvariants, and copy number variations in five studied populations, related to STAR Methods

Locus	Sample	Hapogroup	Allele
DYS447	TCD054	O2a1b1a1a1a	null
	TQH036	Q1b1a	null
DYS448	TML050	D1a1b1a3~	null
	TML122	O2a2b1a1a1a4a2	null
	TCD027	D1a1b1a3~	null
	TCD061	O2a2b	null
DYS549	TML094	O2a2b1a1a1c	null
DYS448	TQH221	D1a1b1a2~	18.2
DYS458	TQH038	J1	18.2
DYS518	TCD013	Q1a1	36.2
	TCD016	Q1a1	37.2
	TCD021	Q1a1	36.2
	TCD046	Q1a1	35.2
	TCD060	Q1a1	37.2
	TCD080	Q1a1	37.2
	TCD101	Q1a1	37.2
	TCD106	Q1a1	37.2
	TQH154	Q1a1	37.2
DYS627	TML149	O2a2a1	21.2
	TCD071	C2a1a2a	20.3
DYS527	TQH017	L	20.2,20.2
DYS385	TML153	O1b1a1	12,18.2
	YLS302	O2a2b1a1a1a4a2a3	13.2,20
DYS19	TQH012	C2a1a2a	16,17
	TQH096	C2a1a2a	16,17
DYS438	TQH001	DE	10,16
DYS458	TQH060	D1a1b1a2~	16,17
DYS460	TQH001	DE	10,11
DYS557	TCD015	O2a2a1	13,14
DYS527	YLS128	O2a1	21,22,23
	YLS188	C2b1b1	19,20,21
DYS385	TML065	O1b1a1a1a1b1	13,14,18
DYF387S1	YLS188	C2b1b1	36,37,39

Table S14. AMOVA Results Based on 33 Chinese Populations, related to STAR Methods

	Groupings	Number of Populations	Number of Groups	Percentage of Variation (%)					
				Among-Groups	Among Populations within Groups	Within-Populations	FCT	FSC	FST
27 Y-STR	Ethnicity	33	7	5.41	1.00	93.59	0.0541	0.0106	0.0641
	Language	33	4	2.34	3.80	93.86	0.0234	0.0390	0.0614
	Altitude	33	3	2.70	4.08	93.22	0.0270	0.0419	0.0678
157 Y-SNP	Ethnicity	33	7	15.32	1.91	82.77	0.1532	0.0226	0.1723
	Language	33	4	8.70	8.54	82.77	0.0870	0.0935	0.1723
	Altitude	33	3	6.65	11.02	82.33	0.0665	0.1180	0.1767
